# SpaMode: A Broadly Applicable Framework for Deciphering Spatial Multi‐Omics Using Multimodal Mixture of Disentangled Experts

**DOI:** 10.1002/advs.75478

**Published:** 2026-05-04

**Authors:** Xubin Zheng, Xinlei Huang, Xiang Zhou, Dian Meng, Ziyue Qiao, Zhiyuan Yuan, Lixin Cheng

**Affiliations:** ^1^ Dongguan Key Laboratory for AI and Dynamical Systems School of Computing and Information Technology Great Bay University Dongguan China; ^2^ Institute of Artificial Intelligence Great Bay University Dongguan China; ^3^ Department of Geriatrics Guangdong Provincial Clinical Research Center for Geriatrics Shenzhen People's Hospital (The First Affiliated Hospital Southern University of Science and Technology The Second Clinical Medical College Jinan University) Shenzhen China; ^4^ Institute of Health Medicine School of Medicine Southern University of Science and Technology Shenzhen China; ^5^ Guangdong Institute of Intelligence Science and Technolog Zhuhai Guangdong China; ^6^ Department of Biomedical Informatics Yong Loo Lin School of Medicine National University of Singapore Singapore Singapore; ^7^ Institute of Science and Technology for Brain‐Inspired Intelligence Fudan University Shanghai China

**Keywords:** Mixture of Experts, Mosaic Integration, Multimodal Disentangling, Spatial multi‐omics

## Abstract

Spatial multi‐omic technologies enable simultaneous multi‐omic profiling within native tissue context, offering unprecedented opportunities to study biological processes and disease. As investigations of tissue spatial architecture gain in complexity, broadly applicable models are required to support vertical (within a section), horizontal (across sections), and mosaic (across distinct omics) integration. Here, we propose SpaMode, a broadly applicable framework designed to accommodate spatial multi‐omic integration for all three modes and four omic types, including transcriptomics, proteomics, epigenomics, and metabolomics. SpaMode disentangles each omics modality into modality‐invariant and modality‐variant distributions to characterize underlying biomolecular commonalities and specificities, and then hierarchically aggregates these distributions to resolve spatial heterogeneity. Horizontal and mosaic integration are unified within the SpaMode framework through multi‐slice joint regularization and translation of missing modalities. We benchmark SpaMode across vertical, horizontal, and mosaic integration, which demonstrate that SpaMode outperforms existing, targeted approaches in all integration settings. Furthermore, SpaMode provides novel insights into how invariant and variant multi‐level biomolecular features contribute divergently to tissue spatial context, offering an interpretable alternative to black‐box neural network. SpaMode provides a general and trustworthy solution for spatial multi‐omic data analysis, paving the way for systematically decoding the complex mechanisms of cellular states and disease evolution in situ.

## Graphical abstract

SpaMode introduces a versatile framework for spatial multi‐omics integration across vertical, horizontal, and mosaic scenarios. By disentangling modality‐invariant and variant features through a mixture‐of‐experts mechanism, it adaptively reconfigures spatially heterogeneous signals. This interpretable approach outperforms existing methods in deciphering complex tissue architectures and enables accurate missing modality translation.

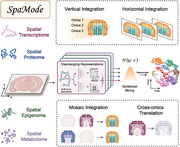

## 1 Introduction

The normal functioning and pathological progression of complex biological tissues are intricately linked to the synergistic interactions of multi‐level biomolecules and the spatial arrangement of cells [1, 2, 3]. Recently, the development of spatially resolved multi‐omics joint sequencing technologies has provided an unprecedented research paradigm for profiling cellular heterogeneity and their multi‐level molecular states within the tissue microenvironment [4, 5, 6]. Existing spatial omics co‐sequencing technologies enable the acquisition of in situ joint transcriptome‐proteome expression profiles (e.g., DBiT‐seq [7], spatial‐CITE‐seq [8], SPOTS [9], SM‐omics [10], stereo‐CITE‐seq [11]), in situ joint transcriptome‐epigenome profiles (e.g., spatial‐RNA‐ATAC‐seq [12], MISAR‐seq [13]), and in situ joint transcriptome‐metabolome profiles (e.g., combining spatial transcriptomics with desorption electrospray ionization [14] and matrix‐assisted laser desorption/ionization [15]). Deciphering spatial tissue domains from these data requires computational approaches capable of constructing a unified representation across multiple spatial omic layers. As the demand for refined and comprehensive analyses of biological processes and diseases grows, it has become necessary to examine tissues from multiple perspectives, including detailed analysis of individual sections, integrative analysis across multiple sections, and assessments in the presence of incomplete omic layers.

Current analyses of tissue domains using spatial multi‐omics mainly fall into three scenarios: vertical integration, horizontal integration, and mosaic integration. Vertical integration involves integrating spatial multi‐omics data within a single tissue section, which requires aggregation of different omic layers and spatial information. Computational methods, such as SpatialGlue [16], COSMOS [17], and PRAGA [18], are specifically designed for spatial multi‐omics data, but they fail to handle complex multi‐section situations. Horizontal integration refers to the co‐integration of spatial multi‐omics data across multiple tissue sections, demanding robustness to batch effects [19, 20, 21]. PRESENT [22] accomplishes this task through jointly capturing the feature relationships across multiple sections, while its reliance on black‐box neural networks [23, 24] results in a lack of interpretability [25, 26, 27]. Mosaic integration entails integrating partial omics modalities, which are incomplete due to budget constraints or technical perturbations [28, 29, 30]. To tackle this, SpaMosaic [31] enhances the representation of the section with missing modalities by constructing a cross‐section heterogeneous graph from a modality‐specific perspective. A significant limitation remains that SpaMosaic fails to bridge the biological insights gap caused by missing modalities, which is a fundamental problem in the mosaic integration scenario.

Comprehensive analyses of biological samples may require a combination of these integration scenarios. However, existing computational methods are designed to target specific integration scenarios. Switching between models for different integration requirements not only results in redundant resources and time but also introduces additional interference stemming from model discrepancies. Therefore, there is a critical need for a general integration framework for spatial multi‐omics that can simultaneously accommodate vertical, horizontal, and mosaic integration. Additionally, we note that existing methods often rely on black‐box neural networks, which leads to a lack of interpretability from a biomolecular perspective.

Here, we propose SpaMode, a broadly applicable framework for spatial multi‐omics analysis, versatile to vertical, horizontal, and mosaic integration. It performs a fine‐grained, disentangled variational encoding to decode modality‐invariant and modality‐variant features correlate with spatial heterogeneity. A mixture of experts weighting mechanism is introduced to adaptively reconfigure these spatially heterogeneous features, providing an interpretable paradigm for spatial domain deciphering. Benchmarking experiments demonstrates the superior performance of SpaMode against existing targeted methods in all the three scenarios. In the vertical integration, SpaMode exhibits significant performance advantages over existing methods and delineated clear and consistent tissue boundaries in spatial domain in five datasets including one simulated and four real datasets. In horizontal integration and mosaic integration tasks, SpaMode shows higher robustness against multi‐slice batch effects and partial modality missingness compared to baseline methods. It is also capable of reconstructing raw count matrices that closely approximated the original source of the missing modality. These results comprehensively validate SpaMode's powerful integration capability for spatial multi‐omics data and its generalization advantages for existing analytical tasks.

## 2 Results

### 2.1 Overview of SpaMode

**FIGURE 1:**
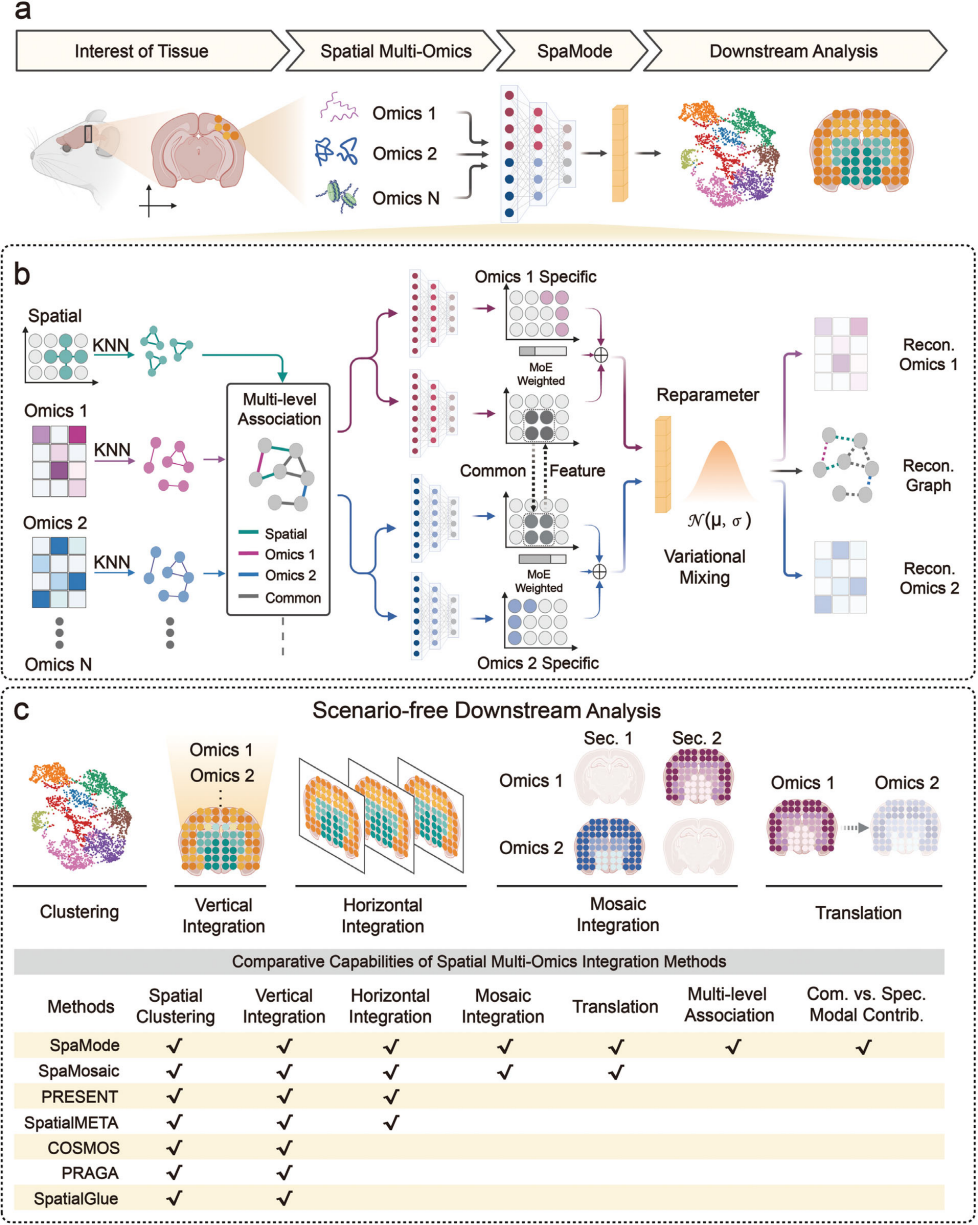
The overview of SpaMode. (a) The workflow spatial multiomics integration. The process starts with multiple spatial multi‐omics datasets (Omics 1 to Omics N) collected from a tissue of interest. SpaMode employs its core model for representation learning, leading to comprehensive integration analysis. (b) The model structure of SpaMode. SpaMode first constructs a multi‐level association network by fusing individual K‐nearest neighbors (KNN) graphs, which capture both molecular‐feature relationships and spatial proximity. Then, SpaMode disentangles the latent representations into two components: modality‐invariant (common) distribution for shared biological features and a modality‐variant (specific) distribution for unique characteristics. A mixture of experts (MoE) weighting scheme guides the weighted combination of the common and specific components to form an integrated modality distribution. Finally, a variational mixing strategy aggregates these specific distributions from all omics modalities into a unified joint distribution for robust representation learning. The model is trained by reconstructing the input omics data and the multi‐level association network. (c) Scenario‐free downstream analysis and comparison with existing methods. SpaMode's joint representation learning facilitates scenario‐free integration analysis, including clustering, vertical integration, horizontal integration, mosaic integration, and cross‐omics translation. The table summarizes the capabilities of SpaMode across various downstream analysis tasks, including spatial clustering, vertical integration, horizontal integration, mosaic integration, translation, and providing unique features like multi‐level omics association and the analysis of common versus specific contributions for each modal compared to other baseline methods.

SpaMode is a broadly applicable framework designed to address diverse spatial omics analysis tasks. Its inputs include omics data from various modalities and their corresponding spatial coordinates. The overall process involves spatial multi‐omics data input, SpaMode's core model for representation learning, and subsequent downstream analysis (Figure 1a).

To simultaneously capture spot relationships at multiple omics and spatial levels, SpaMode first constructs a multi‐level association network by fusing individual K‐nearest neighbors (KNN) graphs, thereby combining both molecular‐feature relationships (omics‐specific KNN graphs) and spatial proximity (spatial KNN graph). Biologically, spatial tissue heterogeneity is defined by consistent cell identities shared across omics, yet refined by distinct regulatory activities unique to each molecular layer. Driven by this insight, SpaMode employs a disentangled graph variational autoencoder framework. Specifically, for each individual omics modality, SpaMode leverages the modality‐specific features and the multi‐level association network to disentangle the latent representations into two biologically meaningful components: a modality‐invariant (common) distribution capturing shared biological states, and a modality‐variant (specific) characterizing unique regulatory features. To adaptively determine the contribution of these common versus specific signals to the local tissue context, SpaMode introduces a mixture of experts weighting (MoEW) scheme. Guided by the MoEW output, the invariant and variant distributions are combined via a weighted linear combination to form an integrated modality distribution. A Variational Mixing strategy then aggregates these specific distributions from all omics modalities into a unified joint distribution for robust representation learning. Latent embedding, sampled from this joint distribution via reparameterization, resolves the spatial heterogeneity of tissues through clustering and subsequent downstream analysis. The model is trained by reconstructing the input omics data and the multi‐level association network (Figure 1b).

SpaMode's joint representation learning facilitates scenario‐free downstream analysis (Figure 1c). This includes scenarios such as: vertical integration (all modalities measured on the same tissue section), horizontal integration (all modalities present across multiple complete sections), and mosaic integration (a challenging real‐world task where certain modalities are missing in specific sections). Furthermore, the learned representations are effective for the translation of missing modalities, accurately filling in unmeasured data based on the integrated spatial context. The comparative table in Figure 1c highlights SpaMode's superior capabilities. It simultaneously supports spatial clustering, vertical, horizontal, and mosaic integration, as well as translation. Notably, SpaMode distinguishes itself by providing insights into multi‐level associations and common versus specific modal contributions, establishing it as a comprehensive tool for spatial multi‐omics analysis.

### 2.2 SpaMode Deciphers Tissue Spatial Heterogeneity

We first evaluated the performance of SpaMode on vertical integration tasks, which integrating multi‐omic data within a single tissue section. In these experiments, we benchmarked SpaMode against existing spatial multi‐omics methods including SpatialGlue [16], COSMOS [17], PRESENT [22], PRAGA [18], SpaMosaic [31], SpatialMETA [32]. To rigorously prevent any ambiguity or bias regarding the benchmark comparisons, we ensured that SpaMode and all comparative algorithms were strictly supplied with the same pre‐processed input feature matrices, dimensionalities, and spatial coordinates across all experiments (see Methods for detailed preprocessing parameters). Five real‐world and one simulated datasets were used for the comparison. These datasets included different omic profiles—transcriptome, proteome, epigenome, and metabolomics—generated using Visium CytAssist, Spatial‐RNA‐ATAC‐seq, and MISAR‐seq sequencing technologies.

#### 2.2.1 SpaMode Integrates Spatial Transcriptomics and Proteomics to Reveal Complementary Patterns

In the joint spatial transcriptomics‐proteomics, i.e., RNA‐ADT joint integration task, we conducted validation experiments on the human lymph node [16] (HLN) dataset, human tonsil [31] (HT) dataset, and the simulated dataset. We generated a suite of simulated datasets incorporating three spatial patterns (F1, F2, F3) across two multi‐modal omics combinations (RNA‐ADT, RNA‐ATAC). The spatial domains within these datasets were designed to include five distinct domains.

**FIGURE 2:**
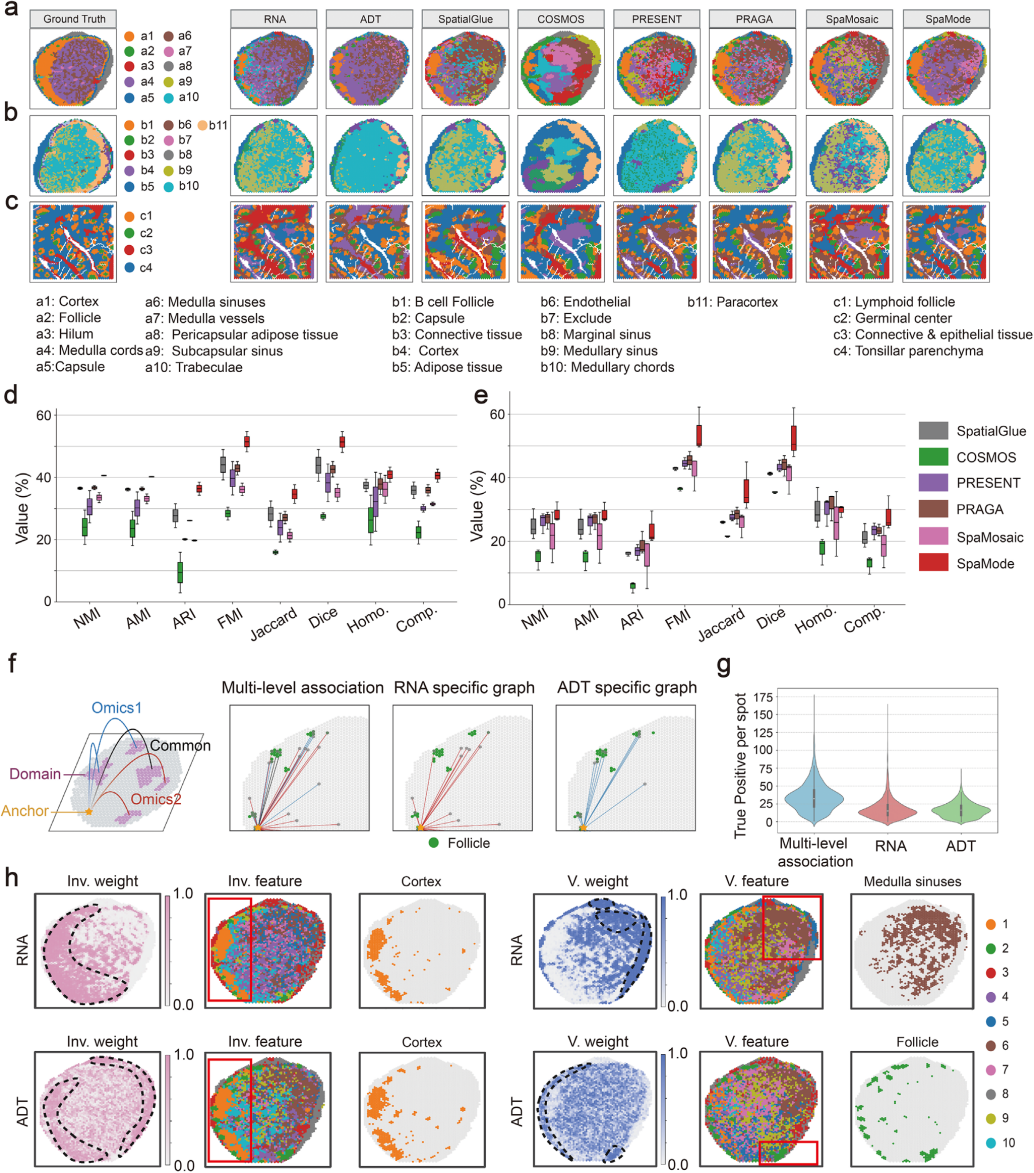
Spatial transcriptomics and proteomics integration. (a–c) Spatial domain of A1 (a) and D1 (b) sections in human lymph node dataset and S3 (c) section in the human tonsil dataset identified SpaMode and other methods compared with manually annotated ground truth. (d) Quantitative comparisons of eight metrics on the human lymph node dataset with five repeated experiments on two sections. (e) Quantitative comparison of eight metrics on the human tonsil node dataset with five repeated experiments on three sections. (f) Spatial visualization of the multi‐level association network, RNA‐only graph, and ADT‐only graph in the follicle region from human lymph node A1 section. (g) The number of true positive connections for each spot in the multi‐level association network, RNA‐only graph, and ADT‐only graph on the human lymph node A1 section. (h) Visualizing the weights of modality‐invariant and modality‐variant features disentangled by SpaMode for RNA and ADT modalities, alongside the associated spatial domain clustering on the human lymph node A1 section.

The visualization results on the HLN A1 section (Figure 2a), HLN D1 section (Figure 2b) and HT S3 section (Figure 2c) consistently demonstrate that SpaMode identifies spatial domains the most consistently to the ground truth compared with the benchmark methods. SpaMode also showed state‐of‐the‐art performance in spatial domain recognition on simulated RNA‐ADT data (Figure S1a) and S1 and S2 slices of human tonsils (Figure S2a–c). Furthermore, we quantified the performance of SpaMode and the benchmark methods from eight metrics including normalized mutual information, adjusted mutual information, adjusted Rand index, Fowlkes‐Mallows index, Jaccard index, Dice's coefficient, homogeneity, and completeness. SpaMode outperformed all the other methods on the HLN dataset (Figure 2d), HT dataset (Figure 2e), and simulated dataset (Figure S1c).

Recognizing that tissue architecture arises from the synergistic interplay of multiple molecular layers, SpaMode constructs a multi‐level association network to integrate these complementary biological signals by fusing feature graphs from distinct omics modalities. To validate the necessity of this design, we visualized the network topology alongside single‐modality graphs within the Follicle region of the HLN A1 dataset (Figure 2f). Visualization reveals that while single‐modality graphs capture limited molecular insights—illustrated by RNA‐specific transcriptomic neighbors (red lines) and ADT‐specific proteomic affinities (blue lines)—the multi‐level association network successfully encapsulates these distinct, long‐distance connections into a unified topology (Figure 2f). By synthesizing these complementary signals, each spot in the multi‐level network establishes connections with a significantly higher number of spots within the same spatial domain compared to single‐modality graphs (Figure 2g), thereby enriching the model with robust inter‐spot relationships. This capacity to capture comprehensive biological relationships was further corroborated by visualizations in the human tonsil dataset (Figure S2d). Thus, encoding omics modalities via this multi‐level association network allows SpaMode to effectively integrate complementary omics features, preserving the integrity of the tissue's complex molecular architecture.

Moreover, the mixture of experts (MoE) weighting of the disentangled invariant and variant features is another critical design applied in SpaMode to capture the association of modules with biological tissue regions (Figure 2h). For the RNA modality, SpaMode primarily focuses more on the invariant features in the cortex and medulla cords, while focusing more on the variant features in the medulla sinuses, pericapsular adipose tissue, and capsule regions. For the ADT modality, SpaMode demonstrates a different pattern of interest, focusing more on the invariant features in the cortex and pericapsular adipose tissue regions, and the variant features in the medulla cords and follicle regions. Additionally, SpaMode captures highly similar invariant features in both RNA and ADT data, for example, both focusing on the cortex. This provides interpretation for SpaMode's accurate identification of the cortex domain. Conversely, the variable features of RNA and ADT focus on different regions: RNA specifically highlights the medulla sinuses, while ADT emphasizes the follicle region. This adaptive association to biological tissue domain enables SpaMode to provide fine‐grained spatial heterogeneity knowledge for latent embeddings, thereby achieving advanced performance on deciphering spatial domains.

We also validated SpaMode's sensitivity on the human lymph node D1 section (Figure S3a,g) to the number of feature neighbors (Figure S3b,c), the number of spatial neighbors (Figure S3d,e), and the number of principal components (Figure S3f,g). Ablation studies were also conducted to verify the effectiveness and contribution of individual modules (Figure S3h,i) and evaluated the impact of different clustering methods [33, 34, 35, 36, 37] on SpaMode's performance (Figure S3j,k).

#### 2.2.2 SpaMode Builds Unified Representations from Spatial Transcriptomics‐Epigenomics Datasets

**FIGURE 3:**
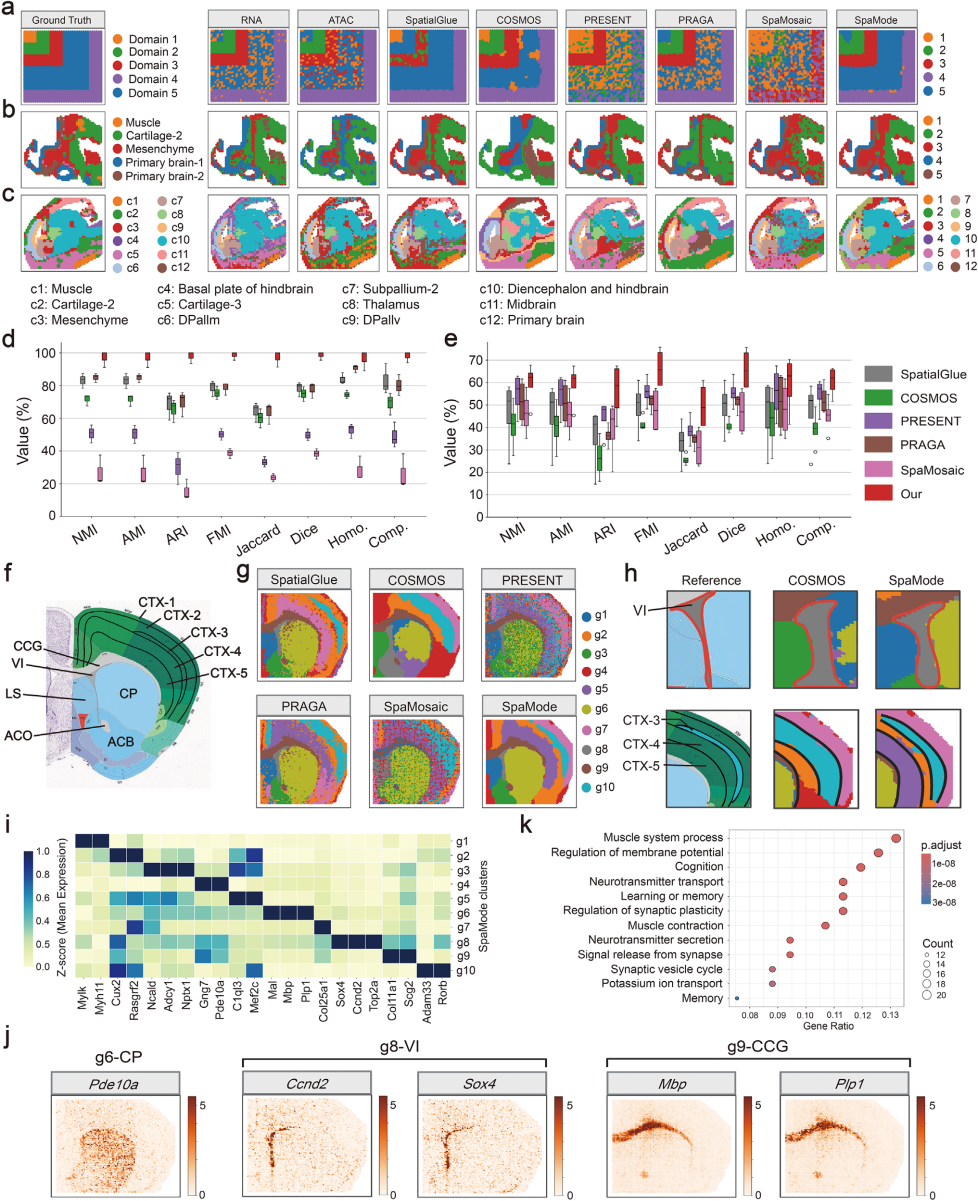
Spatial transcriptomics and epigenomics integration. (a–c) Spatial domain of F3 section in simulated dataset (a), E11.0 section (b), and E15.5 section (c) in mouse embryonic dataset identified SpaMode and other methods compared with manually annotated ground truth. (d) Quantitative comparisons of eight metrics on the simulated dataset experiments on three sections. (e) Quantitative comparison of eight metrics on the mouse embryonic dataset on four sections. (f) Annotation of mouse brain regions referencing the Allen Brain Atlas38. (g) Spatial domains identified by SpaMode and other methods on the mouse brain dataset. (h) Detailed identification of the VI and CTX regions by SpaMode. (i) Heatmap of spatially differentially expressed genes for each domain identified by SpaMode on the mouse brain dataset. (j) Spatial expression visualization of representative differentially expressed genes. (k) Biological process enrichment analysis for differentially expressed genes in cortical regions.

For the spatial transcriptomics‐epigenomics, i.e., RNA‐ATAC joint integration, we initiated the evaluation of SpaMode on the simulated dataset (Figure 3a). SpaMode achieved the best reconstruction performance of all spatial domains across the comparative methods, which can only partially reconstruct spatial domains. We further evaluated SpaMode on practical datasets. The results on the real mouse embryo E11.0 (Figure 3b) and E15.5 dataset (Figure 3c) show that domain identified by SpaMode is also the most consistent with the ground truth compared to the baseline methods (Figure 3d), demonstrating its capability in spatial domain identification. Quantitatively, we compared SpaMode with five benchmarking methods on both simulated dataset (Figure 3d) and mouse embryo dataset (Figure 3e), which consistently demonstrated that SpaMode achieved state‐of‐the‐art quantitative performance. Other sections of the simulated dataset (F1 and F2) and the mouse embryo dataset (E13.5 and E18.5) also illustrated the domain identification of SpaMode (Figures S1b and S4)

We applied spatial smoothing to improve biological coherence among the spatial domain (see Methods for details). Specifically, we compared different smoothing radius ranging from 4 to 8 on the mouse embryo dataset (Figure S5a,b) and found that utilizing an appropriate spatial smoothing radius from 5 to 7 can enhance both clustering quantitative metrics (Figure S5c) and spatial domain continuity (Figure S5d). However, excessive smoothing can lead to neglect of some tissue regions.

Next, we evaluated SpaMode's performance on a higher‐resolution mouse brain spatial RNA‐ATAC co‐assay dataset. Referencing the Allen Brain Atlas [38], we annotated several brain regions (Figure 3f), primarily including the cortex (CTX), genu of the corpus callosum (CCG), lateral ventricle (VL), lateral septal complex (LS), caudoputamen (CP), anterior commissure of olfactory limb (ACO), and nucleus accumbens (ACB). The spatial plots show that SpaMode delineates the brain regions more clearly than the five comparative spatial multi‐omics methods (Figure 3g). Especially, SpaMode demonstrated higher precision in delineating fine‐grained regions such as VL and CTX compared to COSMOS, which also demonstrate good coherence in the brain regions partition (Figure 3k). A detailed partitioning of brain regions is available in Figure S6c.

We also extracted differentially expressed genes (DEGs) for each brain region based on the spatial domain identified by SpaMode (Figure 3i). These marker genes, such as *Mbp*, *Plp1*, and *Mal* in the CCG [39, 40], along with *Rorb* and *Cux2* in the cortex [41, 42, 43], are consistent with biological knowledge (Figure 3j and Figure S6d). Enrichment analysis of biological process on 50 DEGs of cerebral cortex region reveals biological processes belonging to cortex including cognition and memory, further demonstrating the effectiveness on SpaMode in spatial domain identification (Figure 3k). Furthermore, to comprehensively validate the biological functional specificity of the fine‐grained anatomical structures identified by SpaMode, we performed Gene Ontology (GO) enrichment analysis on key brain regions beyond the cortex, including CCG & ACO, CP, VI, ACB, and LS (Figure S7). The results demonstrated high biological consistency: the white matter fiber tract regions were significantly enriched for myelination and glial cell differentiation pathways; the caudoputamen region was enriched for locomotory behavior and synaptic transmission processes; and the lateral septum specifically enriched fear response and cognition‐related functions. These functional enrichment results align closely with the known physiological functions of these brain regions, further confirming the reliability and accuracy of SpaMode in deciphering spatial heterogeneity in complex tissues.

Furthermore, recent studies have demonstrated that appropriate external reference atlases can significantly enhance the analysis of spatial transcriptomics and single‐cell data [44, 45, 46, 47]. To demonstrate SpaMode's architectural flexibility and its capacity to leverage such prior knowledge, we extended our vertical integration analysis on the mouse embryo E18.5 dataset by incorporating an external single‐cell RNA sequencing (scRNA‐seq) reference [48]. Utilizing an E18.5 mouse neocortex scRNA‐seq dataset, we applied soft optimal transport to align the single‐cell and spatial transcriptomic gene expression profiles, obtaining a weighted mapping matrix (Figure S4d). This matrix was used to project the single‐cell expressions into the spatial context, thereby constructing a novel “pseudo‐spatial” modality. Benefiting from SpaMode's scalable design, this pseudo‐spatial modality was seamlessly integrated as a third independent omic layer alongside the existing spatial RNA and ATAC data (Figure S4e). We clustered the single‐cell data using the Leiden algorithm to identify distinct cell populations (Figure S4f) and visualized their multi‐lineage correspondence to the deciphered spatial domains guided by the optimal transport matrix (Figure S4g). Notably, this reference‐guided integration (SpaMode‐SC) further refined the spatial domain deciphering, yielding visually clearer anatomical boundaries (Figure S4h) and achieving an overall enhancement in quantitative clustering metrics compared to the standard dual‐omics integration and baseline methods (Figure S4i). This confirms SpaMode's robust extensibility to incorporate prior knowledge from external single‐cell datasets for enhanced spatial multi‐omics analysis.

#### 2.2.3 SpaMode Resolves Tumor Heterogeneity Using Spatial Transcriptomics‐Metabolomics Data

**FIGURE 4:**
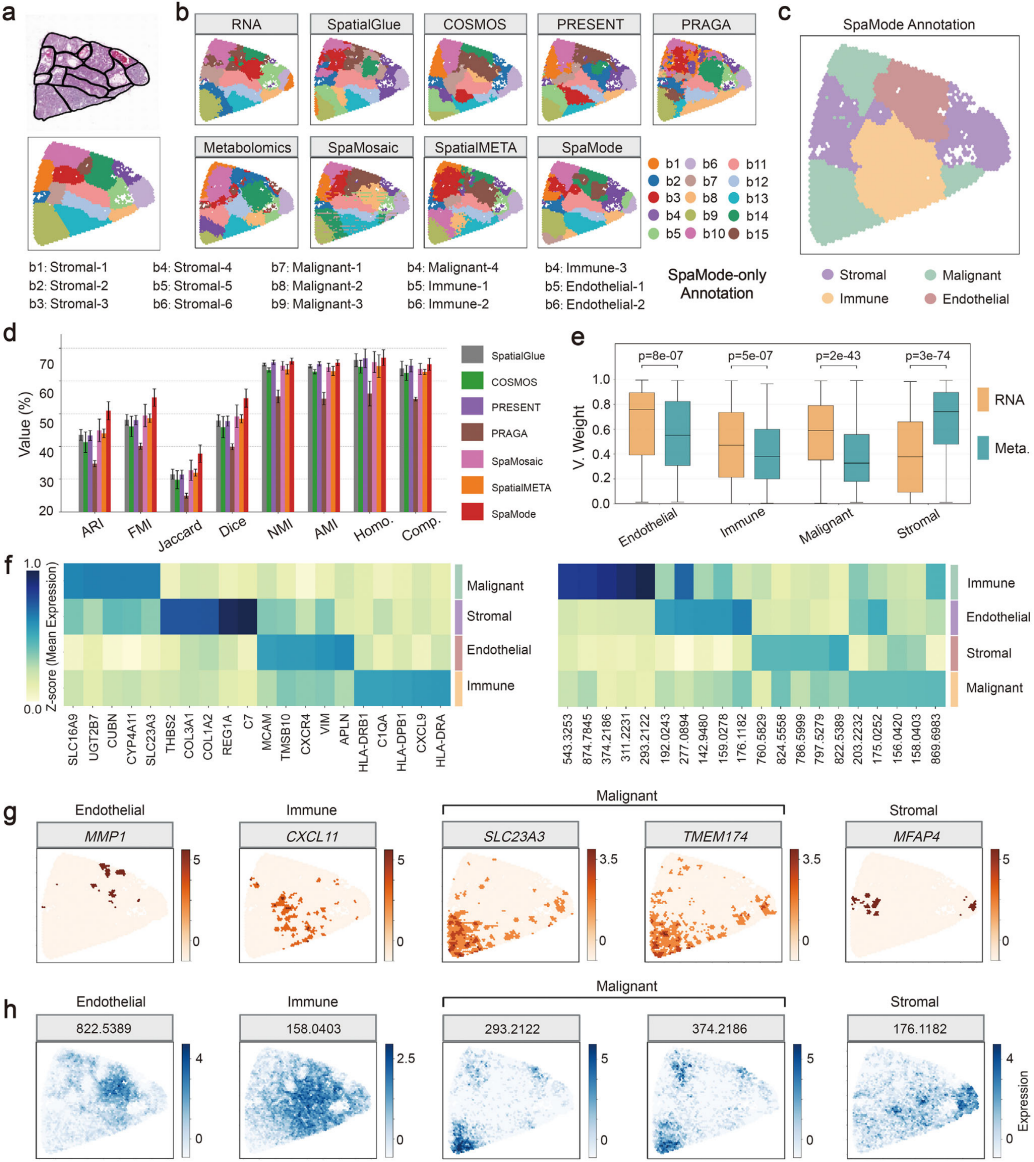
Spatial transcriptomics and metabolomics integration and joint analysis. (a) Manual annotation of spatial domains based on the H&E‐stained image. (b) Spatial domain of human clear cell renal cell carcinoma dataset identified SpaMode and other methods. (c) Functional region annotation based on SpaMode clustering. (d) Quantitative comparisons of integration performance across eight metrics, with error bars representing the variance across clustering numbers ranging from 12 to 20. (e) Visualizing the weights of modality‐invariant and modality‐variant features disentangled by SpaMode for RNA and Metabolite modalities. (f) Heatmap of domain‐specific feature expression showing the Z‐score normalized mean expression of marker genes (RNA) and marker metabolites (Metabolomics) for each of the four main domains. (g) Visualization of domain‐specific gene expression for five exemplary marker genes. (h) Visualization of domain‐specific metabolite expression for five exemplary marker metabolites (represented by their masses) across the four main domains.

We next investigated SpaMode's performance on the joint analysis of spatial transcriptomics‐metabolomics. We utilized a human clear cell renal cell carcinoma dataset (Y7_T) co‐assayed for spatial RNA and metabolites. The domain identification from RNA and metabolite using SpaMode and all benchmark methods demonstrates that SpaMode's domain partition aligns consistently with the manually annotated ground‐truth (Figure 4a,b). SpaMode also provides clearer spatial boundaries compared to other methods (Figure 4b). To characterize the spatial architecture of the tumor microenvironment, we annotated the fifteen SpaMode‐identified clusters into four major functional regions based on canonical molecular signatures: stromal, malignant, endothelial, and immune (Figure 4c).

Quantitatively, SpaMode achieves superior performance over all baseline methods across all eight metrics, including normalized mutual information, adjusted mutual information, adjusted Rand index, Fowlkes‐Mallows index, Jaccard index, Dice's coefficient, homogeneity, and completeness, confirming its robustness in this multi‐omics integration task (Figure 4d). To quantify the modality‐specific contributions to spatial heterogeneity, we visualized the weight bias of the mixture of experts weighting (MoEW) mechanism for disentangled variant features (Figure 4e). Notably, SpaMode revealed a divergent modality reliance: the malignant domain was predominantly driven by transcriptomic variance (RNA‐specific features), whereas the stromal domain exhibited a significantly stronger dependency on metabolic features. This inverse weighting pattern underscores SpaMode's interpretability, demonstrating its capacity to adaptively prioritize the most informative omics layer—genetic regulation in tumor cells versus the metabolic microenvironment in the stroma—to accurately define tissue identity. The heatmap of domain‐specific features shows the Z‐score normalized mean expression of marker genes (RNA) and marker metabolites (Metabolomics) for the four major domains (Figure 4f), confirming the distinct molecular profiles for each region. Finally, we visualized the spatial expression patterns of five exemplary marker genes (*MMP1*, *CXCL11*, *SLC23A3*, *TMEM174*, and *MFAP4*) (Figure 4g) and five exemplary marker metabolites (Figure 4h), which consistently demonstrates the biological relevance of the spatial domains identified by SpaMode.

### 2.3 SpaMode Captures Biological Signals While Eliminating Batch Effects for Multi‐Sections

Next, we evaluated the performance of SpaMode on horizontal integration tasks, which consider the joint integration of multiple sections. We benchmarked four multi‐section joint integration methods, including GraphST [49], STAligner [50], PRESENT [22], and SpaMosaic [31]. These methods employ distinct strategies such as self‐supervised contrastive learning [21, 51, 52], adversarial learning mechanism [53, 54] and Harmony [55] to mitigate batch effects between sections. Among these, both GraphST and STAligner are representative methods tailored for spatial transcriptomics data. While these solutions can mitigate the negative impact of batch effects, they may simultaneously lead to the loss of valuable feature information. Therefore, in SpaMode, we learn batch‐consistent information solely by constructing a cross‐batch feature graph and employing cross‐batch regularization.

**FIGURE 5:**
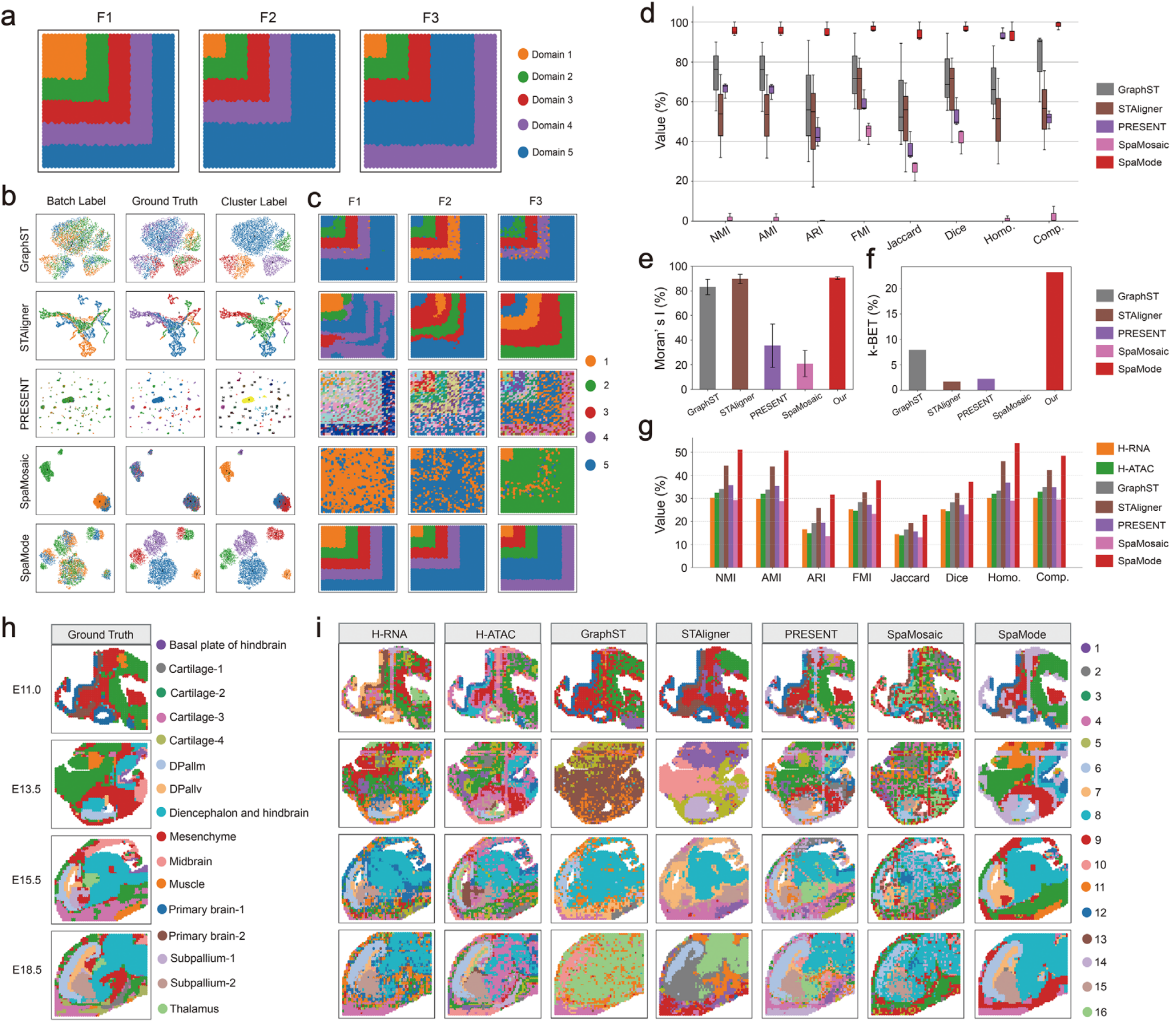
Horizontal integration of multiple spatial multi‐omics slices. (a) Ground truth of F1, F2, F3 sections in the simulated dataset. (b) UMAP visualization of latent encodings generated by SpaMode and benchmark methods, annotated by batch, ground truth, and cluster labels. (c) Visualization of spatial domain identified by SpaMode and comparison methods on the simulated F1, F2, F3 sections. (d) Quantitative clustering metrics of the comparison methods and SpaMode on the simulated F1, F2, F3 sections. (e) Moran's index metrics of the baseline methods and SpaMode on the simulated F1, F2, F3 slices. (f) k‐BET metrics for multi‐batch integration of the baseline methods and SpaMode on the simulated datasets. (g) Joint quantitative clustering metrics of SpaMode and comparison methods and on the mouse embryo dataset. (h) Ground Truth of E11.0, E13.5, E15.5, and E18.5 sections in the mouse embryo dataset. (i) Visualization of spatial domain identified by SpaMode and comparison methods on the joint E11.0, E13.5, E15.5, and E18.5 sections of the mouse embryo dataset.

We first examined SpaMode's horizontal integration capability on a simulated dataset comprising three sections (F1, F2, F3) with shared spatial domains (Figure 5a). The simulated batch effect is achieved through random Gaussian noise. Upon visualizing the UMAP dimensionality reduction of the latent embeddings, we observed that the representations generated by SpaMode effectively mitigate batch effects (Figure 5b). Simultaneously, SpaMode captures cross‐sectionally categorical information almost consistent to the ground truth. Visualization in the spatial context further confirmed SpaMode's capabilities of cross‐slice analysis (Figure 5c). Although there were minor instances of spatial domain misclassification on the F2 and F3 slices, SpaMode successfully delineated clear spatial boundaries for all distinct categories, a result significantly superior to the comparative methods. Quantitatively, SpaMode demonstrated state‐of‐the‐art clustering performance in normalized mutual information, Adjusted mutual information, adjusted Rand index, Fowlkes‐Mallows index, Jaccard index, Dice's coefficient, homogeneity, and completeness (Figure 5d). Furthermore, we calculated Moran's I (Figure 5e) to quantify spatial domain consistency, which demonstrated SpaMode's advantage in stably capturing cross‐section spatial information. To quantify the effectiveness of batch effect removal, we compared SpaMode's k‐BET metric with those of comparing methods (Figure 5f). The significant advantage in k‐BET metrics confirmed SpaMode's robustness to batch effects, despite not employing elaborate or complex designs.

We then conducted a larger‐scale validation experiment using a four‐section RNA‐ATAC dataset (Figure 5h). The visualization results consistently showed that SpaMode was able to partition spatial domains that most closely matched the ground truth across all four sections (Figure 5i). The quantitative metrics results for joint clustering across all sections further demonstrate SpaMode's ability to achieve state‐of‐the‐art joint analysis performance for mouse embryo. These multi‐section joint integration experiments comprehensively demonstrate SpaMode's performance advantage in horizontal integration tasks and its robustness to batch effects.

### 2.4 SpaMode Integrates Mosaic Multi‐Omics and Translates Spatial Expression Profiles

**FIGURE 6:**
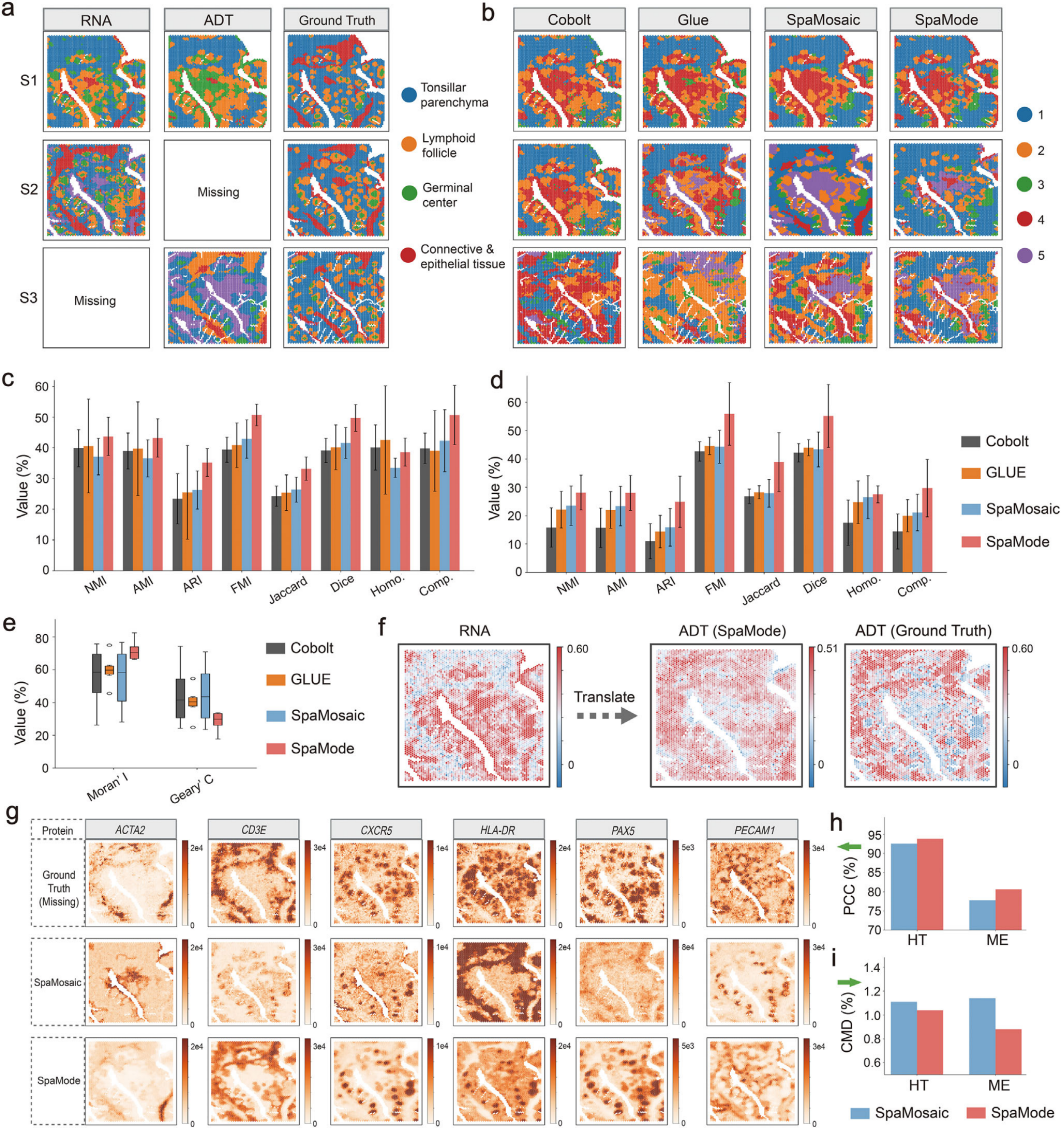
Results of mosaic integration and inference tasks. (a) Input data (RNA and ADT) and manual annotation for three sections of the human tonsil dataset. Sections S2 and S3 contain different omics. (b) Visualization of spatial domain identified by Cobolt, Glue, SpaMosaic, and SpaMode. (c) Quantitative comparison of different methods across seven evaluation metrics on the human tonsil dataset. (d) Quantitative comparison of different methods across eight evaluation metrics on the mouse embryonic dataset. (e) Quantitative comparison of Cobolt, Glue, SpaMosaic, and SpaMode using spatial coherence (including Moran’ I and Geary’ C) on the human tonsil dataset and the mouse embryonic dataset. (f) Spatial distribution of principal components for ADT features translated from RNA via the SpaMode. (g) Comparison of the predicted protein expression translated by SpaMosaic and SpaMode on the human tonsil dataset. (h‐i) Quantitative comparison of SpaMosaic and SpaMode for omics translation on the human tonsil (HT) and mouse embryonic (ME) datasets, evaluated using Pearson correlation coefficient (PCC; h) and cosine metric distance (CMD; i).

SpaMode can also be applied to multi‐section integration with missing omics, i.e., mosaic integration and translation task. First, we conducted tests on a three‐sections human tonsil RNA‐ADT co‐assay dataset. We assumed that the S1 section had all modalities available, the S2 section was missing the ADT modality, and the S3 section was missing the RNA modality (Figure 6a). We compared SpaMode with two state‐of‐the‐art well‐known single‐cell mosaic integration methods (Cobolt [56] and GLUE [57]) and the recent spatial mosaic integration method SpaMosaic. The spatial domain visualization demonstrate that SpaMode can perform low‐noise, high‐accuracy spatial domain partitioning even with incomplete modalities (Figure 6b). Similar experiments were also validated on the mouse embryo RNA‐ATAC co‐assay dataset (Figure S8a,b). Quantitatively, SpaMode exhibited state‐of‐the‐art performance on all 8 quantitative metrics, both in the human tonsil tissue (Figure 6c) and the mouse embryo tissue (Figure 6d). We compared the Moran's I and Geary's C values of spatial domains integrated by SpaMode and baseline methods to assess spatial self‐correlation. As shown in Figure 6e, SpaMode achieved the highest Moran's I and the lowest Geary's C, which demonstrates SpaMode's ability to identify low‐noise spatial regions.

SpaMode addresses the challenging mosaic integration task by converting it into a more readily manageable vertical integration task through the approximation of principal component (PC) features for the missing omics modalities. We visualized SpaMode's approximation of the missing ADT modality's PC features in the S2 section of the human tonsil dataset (Figure 6h). The results prove that the PC features approximated by SpaMode exhibit spatial patterns similar to the original ADT features (Figure 6g), providing valuable information for the analysis of slices with missing modalities.

Then, we evaluated SpaMode on the translation of the missing modality's raw data. We visualized the spatial expression patterns translated by SpaMode and SpaMosaic on the human tonsil dataset (Figure 6g). It can be observed that the expressions translated by SpaMode more closely resembles the true spatial expression patterns compared to the results from SpaMosaic. Similar experimental results were also observed in the mouse embryo tissue dataset (Figure S8c). Quantitatively, SpaMode exhibited superior performance over SpaMosaic, as evidenced by its higher Pearson correlation coefficient (PCC; Figure 6h) and lower correlation matrix distance (CMD; Figure 6i), which indicates that SpaMode's translation outcomes more accurately reflect the true gene expression patterns.

## 3 Discussion

SpaMode is a general and interpretable model for spatial multi‐omic deciphering. It comprehensively characterizes the feature distributions of multiple omics within a spatial context by constructing a multi‐level association network and employing fine‐grained, disentangled variational learning. We benchmarked SpaMode across three common spatial multi‐omic scenarios—vertical integration, horizontal integration, and mosaic integration—using four data settings (simulated data, joint RNA‐ADT, and joint RNA‐ATAC, joint RNA‐metabolite) to demonstrate its state‐of‐the‐art performance and generalizability. In vertical integration tasks, SpaMode demonstrated superior spatial awareness and state‐of‐the‐art quantitative performance compared to seven baseline methods across five manually annotated datasets comprising fifteen tissue sections. On a high‐resolution mouse brain dataset without manual annotations, SpaMode identified spatial domains that are comparable to or even more refined than the Allen Brain Atlas, providing a fine‐grained perspective for investigating the spatial regulatory mechanisms of biological molecules. For horizontal integration, SpaMode utilizes an end‐to‐end approach to jointly encode multiple tissue slices. It demonstrates consistent spatial domain identification across slices and robustness to batch effects, while accurately preserving the specific spatial patterns within each individual section. In mosaic integration tasks, SpaMode approximates the principal component features of missing modalities by combining reconstruction and inference. By effectively capturing features from the missing modalities, SpaMode demonstrates superior mosaic integration capability compared to SpaMosaic, a model specifically designed for this task. Furthermore, SpaMode also reconstructs the raw data matrix of the missing modalities, successfully recovering spatial expression patterns similar to the ground truth.

Compared to existing spatial multi‐omic models designed for specific scenarios, SpaMode presents four unique advantages: 1) SpaMode is a general model capable of handling diverse task scenarios, not being confined to a specific task design. 2) multi‐level association network constructed by SpaMode breaks the information barriers between different omics modalities for the first time. This enables SpaMode to consider global biomolecular relationships regardless of which specific omics modality is being encoded. 3) The combination of fine‐grained disentangled variational encoding and two‐stage distribution weighted mixing provides interpretability to the encoding process of SpaMode. Through the spatial visualization of weights for invariant and variant distributions and their resulting clusters in our experiments, we can clearly explain the connections between different encoding modules and biological structures. 4) The design, which reconstructs principal component features and count matrices for missing modalities, enables SpaMode to capture characteristic information from the absent modality. This offers a new approach for reducing the costs of subsequent studies that rely on joint spatial multi‐omic sequencing of adjacent tissue sections.

An inherent limitation of biological data is that raw sequencing data can be extremely sparse, even approaching near‐binary properties. While SpaMode employs standard Gaussian priors, it is crucial to note that this assumption is applied to the low‐dimensional, continuous latent embeddings rather than directly to the sparse raw matrices. During the encoding process, the high‐dimensional sparse signals are aggregated into dense, continuous representations. In this reduced space, the extreme sparsity is effectively mitigated, and the data reflect continuous biological states or gradients. The Gaussian prior serves as a necessary regularizer to enforce a smooth latent manifold, ensuring stable variational mixing and robust disentanglement of modality‐invariant and variant features. Therefore, modeling these dense, aggregated representations with a Gaussian distribution is theoretically sound. Nevertheless, exploring discrete or zero‐inflated priors explicitly tailored for such extreme sparsity in raw data represents a promising theoretical direction for future research.

To establish the theoretical boundaries of the framework, the efficacy of SpaMode's disentanglement relies on the presence of underlying biological synergies between modalities. In an extreme hypothetical scenario where input modalities capture entirely orthogonal or non‐overlapping biological signals, the modality‐invariant distributions would inherently capture minimal shared context. Under these conditions, SpaMode's mixture of experts weighting (MoEW) mechanism would robustly adapt by assigning near‐maximum weights to the modality‐variant distributions to preserve distinct modal identities. While the framework remains mathematically stable via this adaptive routing, the analytical benefits of cross‐modality synergy would naturally be limited by the biological data itself.

As spatial omics sequencing technologies continue to advance, the generation of joint spatial multi‐omic sequencing data incorporating a greater number of modalities is an inevitable trend. With this in mind, we have designed SpaMode with a modular and flexible architecture, enabling it to support an arbitrary number of omics modalities as input. Furthermore, the fine‐grained disentangled variational encoding design is not limited to omics data features. SpaMode can be easily extended to incorporate other data modalities, such as H&E stained images [58], with only minor code modifications.

The cost of spatial multi‐omics sequencing on serial sections also presents a significant obstacle for future research. Existing mosaic integration scenarios only simulate the problem of missing modalities in some sections while adjacent sections remain complete. They do not yet account for the more complex consideration where the common modalities in adjacent sections are also missing. Therefore, we plan to further enhance SpaMode to handle the joint integration and inference of unpaired data, offering the potential to reduce the costs associated with sequencing serial sections.

With the advancement of spatial multi‐omic sequencing technologies, we expect SpaMode to be a universal analytical tool capable of addressing diverse scales, data compositions, and task requirements. It has the potential to mine valuable biomarkers, potential regulatory relationships, and spatiotemporal dynamics from spatial multi‐omics data. Furthermore, it may provide new solutions for unveiling pathological mechanisms and the evolutionary processes of the tumor microenvironment.

## 4 Methods

### 4.1 Data Preparation

#### 4.1.1 Simulated Datasets

Following the work of Townes et al. [59], we generated simulated datasets using a factor model‐based framework. For the spatial structure, we adopted three preset, approximate geometric patterns of “multi‐level tissue boundaries” as the ground truth for the latent spatial patterns. Each spatial pattern consists of 1,296 spatial spots. We assigned an independent number of features for each modality; for instance, we set 600 genes for the RNA modality, 350 features for the protein modality, and simulated 1000 chromatin accessibility regions (peaks) for the ATAC modality.

At the data generation level, different statistical distributions were employed for different modalities (Figure S1d). For the RNA modality, we sampled from a zero‐inflated negative binomial (ZINB) distribution. For the protein modality, we generated simulated data following a negative binomial (NB) distribution. For the ATAC modality, we sampled from a binomial distribution (specifically, a Bernoulli distribution) to simulate the biological state of chromatin regions being either “open” or “closed.” The probability of a peak being open at a specific spatial spot was also calculated by the mean formula µ, with a truncation function applied to ensure the probability value did not exceed 1.

To model variations between different experimental batches, we additionally generated a version of the simulated data with batch effects. This was achieved by introducing a random Gaussian noise into different “slices” to simulate the offset caused by an additive batch effect.

#### 4.1.2 Human Lymph Node (HLN) Dataset

The human lymph node (HLN) dataset was generated from two consecutive, 5‐µm thick, formalin‐fixed paraffin‐embedded (FFPE) tissue sections. This dataset was created using the 10x Genomics CytAssist Visium platform, enabling the simultaneous analysis of two data modalities: spatial transcriptomics and spatial proteomics. For the analysis, gene expression was profiled using the Visium Human Transcriptome Probe Set v2.0, while protein detection was performed with a 35‐antibody Human FFPE Immuno‐oncology panel. All resulting libraries were sequenced using high‐throughput Illumina NovaSeq S2 PE50 sequencing technology. In terms of data analysis, the data were processed using the Space Ranger software. Subsequently, based on high‐resolution microscopy images, the anatomical structures of the lymph node were manually annotated within the Loupe Browser software, thereby integrating precise molecular information with its histological context.

#### 4.1.3 Human Tonsil (HT) Dataset

This human tonsil dataset was established for the evaluation of various analytical methods, including SpaMosaic. The dataset comprises three tissue sections, from which multi‐modal data for spatial transcriptomics (RNA) and protein (ADT) were simultaneously acquired using the 10x Genomics Visium platform. Furthermore, the study incorporates manual annotations by experts on H&E stained images to support subsequent analyses. This provides a precise spatial context for investigating the complex immune microenvironment structures within the tonsil, such as B‐cell follicles and germinal centers.

#### 4.1.4 Mouse Embryonic (MISAR) Dataset

The mouse embryonic dataset [13] originates from a study on mouse brain development that utilized MISAR‐seq technology, providing paired spatial gene expression and spatial chromatin accessibility data. The research covers four key, manually annotated developmental stages: embryonic day 11.0 (E11.0) with 1,258 spots, E13.5 with 1,777 spots, E15.5 with 1,949 spots, and E18.5 with 2,129 spots. For the epigenetic analysis, the research team used the MACS2 software to identify chromatin accessibility regions (peaks) in each sample, which yielded a range of approximately 240,000 to 290,000 peaks per sample.

#### 4.1.5 Mouse Brain (MB) Dataset

The mouse brain dataset [12] originates from a study on juvenile (P22) mouse brain tissue, which aimed to simultaneously resolve its spatial epigenome and transcriptome. This multi‐modal dataset contains spatial chromatin accessibility data and spatial transcriptomics data from over 9000 sites on a coronal section of the mouse brain. We followed the preprocessing workflow of the original authors to process this data and obtained 30D principal component features.

#### 4.1.6 Clear Cell Renal Cell Carcinoma (ccRCC) Dataset

The ccRCC (Y7_T) Dataset was generated for an investigation into the metabolic reprogramming associated with renal cell carcinoma progression, comprising paired spatial transcriptomics (ST) and spatial metabolomics (SM) data. The ST data was generated using the 10x Genomics Visium platform, while the SM data was derived from either MALDI or DESI platforms. Crucially, as the data originated from adjacent tissue sections, the raw data presented significant disparities in spatial morphology and resolution, such as the Y7_T sample having 2,018 ST spots compared to 10,145 SM spots. In preparation for analysis, this dataset required the SpatialMETA framework to perform alignment and reassignment using the K‐nearest neighbor (KNN) approach to unify the spatial resolution by aggregating the denser SM data to the lower resolution ST spots. The processed and integrated data were then utilized to characterize immune spatial clusters, notably revealing a sub‐cluster enriched for upregulated lipid metabolic pathways.

#### 4.1.7 Mouse Neocortex Single‐Cell Reference Dataset

The single‐cell RNA sequencing (scRNA‐seq) reference dataset utilized for the extended integration analysis originates from a study investigating progenitor cell diversity in the developing mouse neocortex [48]. The original data was generated using droplet‐based scRNA‐Seq (Drop‐Seq) from mouse dorsal forebrain tissues across multiple embryonic stages. For our optimal transport and pseudo‐spatial modality construction, we specifically extracted the data corresponding to the embryonic day 18.5 (E18.5) stage. Following the quality control and preprocessing guidelines detailed in the original study, low‐quality cells and doublets were filtered out, resulting in a robust reference subset comprising 1,022 high‐quality cells. This processed scRNA‐seq data was then mapped to the spatial transcriptomics spots of the matched E18.5 MISAR‐seq dataset to validate SpaMode's extensibility.

#### 4.1.8 Data Preprocessing

To ensure fair benchmarking and consistent inputs across all comparative algorithms, we applied a strictly unified preprocessing pipeline to all spatial multi‐omics datasets. For the transcriptomics (RNA) modality, raw count matrices were filtered to remove genes expressed in fewer than 10 cells. The top 3000 highly variable genes were selected, normalized to a target sum of 10 000, log1p‐transformed, and scaled. Principal component analysis (PCA) was then applied to extract the top 30 principal components. For the proteomics (ADT) modality, raw counts were normalized using the centered log‐ratio (CLR) transformation, followed by PCA to extract the top 30 components. For the epigenomics (ATAC) modality, peak‐by‐cell matrices were transformed using Term Frequency‐Inverse Document Frequency (TF‐IDF) and reduced to the top 50 principal components via PCA. All spatially‐aware baseline methods were identically provided with these exact same reduced‐dimensional embeddings and raw spatial coordinates.

### 4.2 Framework of SpaMode

SpaMode is a hybrid encoding model for spatial multi‐omics, built upon a graph variational autoencoder and a disentangled expert model. To facilitate inter‐modal information interaction, SpaMode constructs a multi‐level association network by combining the inter‐feature relationships and spatial neighborhoods from all omics modalities. Based on this multi‐level association network, SpaMode performs disentangled variational encoding for each specific modality, separating its features into a modality‐invariant distribution and a modality‐variant distribution. These distributions are then adaptively weighted using a mixture of experts weighting mechanism (MoEW) to generate an integrated modality distribution. The specific distributions from different modalities are subsequently fused via a product of experts (PoE) to obtain a joint distribution. Finally, the joint encoding is acquired from this joint distribution through a re‐sampling technique.

The core framework of SpaMode, as described above, can be divided into four primary steps: 1) multi‐level association network construction, 2) disentangled representation, 3) modality‐specific distribution generation, and 4) multi‐omics variational mixing. Below we describe these steps formally using mathematical formulations.

#### 4.2.1 Multi‐Level Association Network Construction

Given a set of principal component features for different omics modalities, denoted as $X_{M}\in R^{N\times D_{M}}$, where $M\in \{M_{1},M_{2},\text{…},M_{m}\}$ is the modality indicator, *N* is the number of spots, and *D_M_* is the feature dimension for modality *M*. The spots also have corresponding set of spatial coordinates 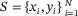. SpaMode first constructs a feature graph for each modality using the k‐nearest neighbor (k‐NN) principle based on Euclidean distance in the feature space. This graph is defined as a combination of features and an adjacency matrix $G_{M}=\left(X_{M},A_{M}\right)$, where $A_{M}\in {\left\{0,1\right\}}^{N\times N}$ is a binary adjacency matrix. ***A***_***i***, ***j***_ is 1 when spot *j* lies among the *k* closest neighbors of spot *i* in feature space, and 0 otherwise. Similarly, the spatial graph is constructed based on Euclidean distance between the spatial coordinates *S* using the k‐NN principle, formulated as $G_{S}=\left(E,A_{S}\right)$. Here, the node features are represented by *E*, an identity matrix, as the spatial graph is used only to model spatial neighborhood relationships and does not involve omics features.

To address the information barrier between modalities, SpaMode construct a multi‐level association network that encompasses information from all modalities and their spatial neighborhood relationships. Specifically, SpaMode calculates an inter‐modal shared feature subgraph $\;G_{\mathrm{share}}=\left(E,A_{\mathrm{share}}\right)$, where:

$$\;A_{\mathrm{share}}=A_{M_{1}}\;\cap A_{M_{2}}\cap \text{…}\cap A_{M_{m}}$$

The modality‐specific graph weights are calculated via the cosine similarity between the modality‐specific feature graph and the shared feature subgraph:

$$\;w_{M}^{G}=\frac{A_{M}\cdot A_{\mathrm{share}}}{∥A_{M}∥∥A_{\mathrm{share}}∥}\;$$

The multi‐level association network $G_{mlan}=\left(E,A_{mlan}\right)$ is constructed through a weighted fusion of the feature graphs from all modalities while also incorporating the spatial graph:

$$\;A_{mlan}=\sum_{M\in \left\{M_{1},M_{2},\text{…},M_{m}\right\}}w_{M}^{G}A_{M}+A_{S}.$$

The multi‐level association network integrates both the intra‐modal feature relationships from all omics modalities and spatial neighborhood relationships. This unified representation enables SpaMode to consider context information from other modalities when encoding each modality independently, thereby breaking the information barrier between omics.

#### 4.2.2 Fine‐Grained Disentangled Representation

Building upon the constructed multi‐level association network, SpaMode performs a multi‐expert disentangled representation for each specific modality. Specifically, SpaMode applies a graph convolutional network [60] (GCN) model as an encoder to generate specific embeddings $H_{M}\in R^{N\times D_{H}}$ for each modality based on the multi‐level association network and the modality's own features:

$$\;H_{M}={\mathrm{GCN}}_{M}^{en}\;\left(X_{M},A_{mlan}\right)$$

Here, *D*_H_ is the dimensionality of the hidden embedding space, and ${\mathrm{GCN}}_{M}^{en}$ denotes the GCN encoder tailored to modality *M*. Furthermore, for each specific modality, SpaMode employs two expert models: an invariant feature expert and a variant feature expert. These are tasked with learning a modality‐invariant distribution $p_{\phi_{\mathrm{inv}}}^{M,\;inv}$ and a modality‐variant distribution $p_{\phi_{v}}^{M,\;v}$, respectively. Both distributions are assumed Gaussian priors based on disentanglement of the modality embeddings ***H**_M_*:

$$p_{\phi_{inv}}^{M,\;inv}\;\left(Z_{M,\;inv}|H_{M}\right)=N\left(Z_{M,\;inv}|\mu_{\phi_{inv}}\left(H_{M}\right),\;diag\left(\sigma_{\phi_{inv}}^{2}\left(H_{M}\right)\right)\right)$$

$$p_{\phi_{v}}^{M,\;v}\;\left(Z_{M,\;v}|H_{M}\right)=N\left(Z_{M,\;inv}|\mu_{\phi_{v}}\left(H_{M}\right),\;diag\left(\sigma_{\phi_{v}}^{2}\left(H_{M}\right)\right)\right)$$

here, ***Z**_M_* represents the latent code, and N(·) denotes a Gaussian distribution. The terms ***μ*** and ***σ*** ^2^ are the mean and variance of this Gaussian distribution, respectively, which are output by the expert models. ϕ represents the parameters of the expert model, and the subscripts *inv* and *v* are used to denote the invariant and variant experts, respectively.

To enable effective disentanglement of invariant and variant distributions, SpaMode construct a discriminator with a multi‐layer perceptron (MLP) architecture to perform adversarial learning independently from the encoding process. This process aims to clearly separate the modality‐common features from the modality‐specific features:

$$\begin{array}{cc} \;L_{Adv} & =-\sum_{M\in \left\{M_{1},M_{2},\text{…},M_{m}\right\}}\mathrm{MLP}\left(Z_{M,\;v}\right)\log Y_{M}\\ & \;+\sum_{M\in \left\{M_{1},M_{2},\text{…},M_{m}\right\}}\mathrm{MLP}\left(Z_{M,inv}\right)\log Y_{M} \end{array}$$

here, MLP(·) represents the multi‐layer perceptron (MLP) discriminator, *Y_m_* denotes the modality label. Both the variant and invariant encodings are obtained by sampling from their respective distributions using the reparameterization trick:

$$\;Z_{M,v}=\mu_{\phi_{v}}\left(H_{M}\right)+\sigma_{\phi_{v}}^{2}\left(H_{M}\right)\cdot \eta_{v}$$

$$\;Z_{M,inv}=\mu_{\phi_{inv}}\left(H_{M}\right)+\sigma_{\phi_{inv}}^{2}\left(H_{M}\right)\cdot \eta_{inv}\;$$

where $\eta_{inv}∼N\left(0,1\right)$ and $\eta_{v}∼N\left(0,1\right)$ are random variable sampled from a standard normal distribution. To prevent excessive loss of modality‐specific information, SpaMode conducts adversarial learning by replacing the variant encoding with a modality‐specific encoding during implementation.

#### 4.2.3 Integrated Modality Distribution Generation

In order to preserve information integrity while accommodating the differential requirements of invariant and variant features across diverse spatial spots, SpaMode generates integrated modality distributions by weighted mixing of invariant and variant distributions through a Mixture‐of‐Experts Weights (MoEW) mechanism. The MoEW mechanism is formulated as follows:

$$\;W_{\mathrm{MoEW}}=\mathrm{softmax}\left(H_{M}W_{1}+\eta_{2}\left(\mathrm{softplus}\left(H_{M}W_{2}\right)+\varepsilon \right)\right)$$

here, *W*_MoEW_ = [*w*_inv_,*w_v_*] ​ represent the weights for the invariant and variant experts, respectively. $W_{1}\in R^{D_{H}\times 2}$​ and $W_{2}\in R^{D_{H}\times 2}$​ are learnable parameters, while $\eta_{2}∼N\left(0,1\right)$ is noise sampled from a Gaussian distribution. The term ***ε*** is a numerical stability term with a fixed value of 0.01, used to ensure the noise standard deviation does not approach zero.

To prevent the Mixture of Experts weights from becoming overly biased towards a single expert, SpaMode follows the standard MoE framework and introduce a balancing loss:

$$\;I_{\mathrm{MoEW}}=\left[\sum_{i=1}^{N}w_{inv,i},\sum_{i=1}^{N}w_{v,i}\right]\;$$

$$\;L_{\mathrm{Balancing}}=\frac{Var\left(I_{\mathrm{MoEW}}\right)}{Mean\left(I_{\mathrm{MoEW}}\right)}\;$$

Based on the MoEW, SpaMode generates the modality distribution *p^M^* (***Z**_M_*) by smoothly performing a weighted mixture of the invariant and variant distributions:

$$\;\mu_{M}=w_{\mathrm{inv}}\;\mu_{\phi_{\mathrm{inv}}}\left(H_{M}\right)+w_{v}\mu_{\phi_{v}}\left(H_{M}\right)$$

$$\;\sigma_{M}^{2}=e^{w_{\mathrm{inv}}\log\sigma_{\phi_{\mathrm{inv}}}^{2}\left(H_{M}\right)+w_{v}\log\sigma_{\phi_{v}}^{2}\left(H_{M}\right)\;\;}\;$$

$$p^{M}\;\left(Z_{M}\right)=N\left(Z_{M}|\mu_{M,\;}\mathrm{diag}\left(\sigma_{M}^{2}\right)\right)$$

To regularize the variational process, SpaMode follow the standard variational autoencoder (VAE) framework and formulate a loss term based on the Kullback‐Leibler divergence between the prior Gaussian distribution and the modality distribution:

$$H_{KL}\;\left(P||Q\right)=\int P\left(x\right)\log\left(\frac{P\left(x\right)}{Q\left(x\right)}\right)dx\;$$

$$\begin{array}{cc} \;L_{KL} & =\frac{1}{m}\;\sum_{M\in \left\{M_{1},M_{2},\text{…},M_{m}\right\}}H_{KL}\left(N\left(Z_{M}|\mu_{M,\;}\mathrm{diag}\left(\sigma_{M}^{2}\right)\right)\left|\right|N\left(0,I\right)\right)\\ & =\frac{1}{2m}\;\sum_{M\in \left\{M_{1},M_{2},\text{…},M_{m}\right\}}\left(\mu_{M}^{2}+\sigma_{M}^{2}-\log\sigma_{M}^{2}-1\right) \end{array}$$

#### 4.2.4 Multi‐Omics Variational Mixing

After obtaining the specific distribution for each omics modality, SpaMode utilizes the Product of Experts (PoE) mixing technique to fuse the distributions from the different modalities into a joint distribution:

$$\mu_{\mathrm{Latent}}=\frac{\sum_{M\in \left\{M_{1},M_{2}\right\}}\mu_{M}⊙{\left(\sigma_{M}^{2}\right)}^{-1}}{\sum_{M\in \left\{M_{1},M_{2}\right\}}\left({\left(\sigma_{M}^{2}\right)}^{-1}\right)}\;$$

$$\sigma_{\mathrm{Latent}}^{2}=\frac{1}{\sum_{M\in \left\{M_{1},M_{2}\right\}}\left({\left(\sigma_{M}^{2}\right)}^{-1}\right)}\;$$

where ⊙ is Hadamard Product. The comprehensive distribution after mixing is expressed as

$$p=N\left(Z|\mu_{\mathrm{Latent}},\mathrm{diag}\left(\sigma_{\mathrm{Latent}}^{2}\right)\right)$$

The latent encoding is obtained by sampling from the joint distribution using the reparameterization trick:

$$Z\;=\mu_{\mathrm{Latent}}+\eta_{3}⊙\sigma_{\mathrm{Latent}}$$

where $\eta_{3}∼N\left(0,1\right)$ is sampled from a standard normal distribution. Spatial domain labels are assigned to each spot by clustering the latent encodings. To obtain a low‐noise spatial domain, SpaMode employs a spatial rectification mechanism to denoise the spatial domain labels. For each spot, SpaMode considers neighboring spots within a radius of SR and corrects the current spot's label if the number of neighbors with a certain label exceeds a threshold γ. Empirically, the threshold γ is set to represent a majority of the neighbors within the SR to ensure that the label correction is driven by a dominant surrounding spatial context, thereby preventing over‐smoothing of fine‐grained tissue boundaries. To confirm the robustness of this setting, we performed a parameter sensitivity analysis for γ in mouse embryonic dataset E15.5 (Figure S5e,f). We found that while a low γ value risks excessive smoothing, SpaMode's performance and spatial visualization remain robust across a moderate to high range (e.g., γ between 3 and 5 when SR = 6, corresponding to roughly 50%–80% of neighbors). Based on these results, we establish a conservative default threshold of roughly 70% of neighbors.

It is worth noting that spatial smoothing is not a mandatory preprocessing requirement for all inputs. Rather, we applied it here as a targeted intervention specifically designed to mitigate the elevated technical noise inherent in certain datasets, such as the mouse embryo sections, thereby improving biological coherence among the spatial domains.

To ensure that valuable modality‐specific information is preserved during the encoding process, SpaMode constructs a modality‐specific decoder based on a graph convolutional network (GCN) model and the spatial neighborhood graph. This decoder reconstructs the latent encoding back into the specific features $\hat{\;X_{M}}$ for each modality:

$$\;\hat{X_{M}}={\mathrm{GCN}}_{M}^{de}\;\left(Z,A_{S}\right)$$

SpaMode calculates the reconstruction loss using the L2 norm:

$$\;L_{\mathrm{recon}}=\sum_{M\in \left\{M_{1},M_{2},\text{…},M_{m}\right\}}w_{M}{∥X_{M}-\hat{X_{M}}∥}_{2}^{2}$$

where *w_M_* is the reconstruction loss weight of modality *M*, set to 1 by default. The reconstruction loss and the Kullback‐Leibler divergence jointly constitute the total model loss, which corresponds to the negative Evidence Lower Bound (ELBO).

$$\;L_{\mathrm{ELBO}}=L_{\mathrm{recon}}+w_{KL}L_{KL}$$

where *w_KL_* is the Kullback‐Leibler divergence weight, which is set to 1E‐6 in practice. Additionally, to preserve the correlations between spots, we have incorporated a graph reconstruction design into SpaMode. Specifically, SpaMode constructs a binary discriminator that takes the latent encodings as input to determine whether an edge exists between two spots. The graph reconstruction loss is then formulated using the universal graph as the reference:

$$\;L_{\mathrm{graph}}=-\sum_{i\neq j,i\in \left\{1,2,\text{…},N\right\},j\in \left\{1,2,\text{…},N\right\}}\psi \left(Z_{i},Z_{j}\right)\log A_{pan,i,j}$$

where ψ(·, ·) is a discriminator used to determine whether there is an edge between two spots based on the latent encoding. Overall, the training process of SpaMode is supervised by four distinct loss components:

$$\;L_{\mathrm{Total}}=L_{\mathrm{ELBO}}+w_{\mathrm{graph}}L_{\mathrm{graph}}+w_{\mathrm{Balancing}}L_{\mathrm{Balancing}}+w_{\mathrm{Adv}}L_{\mathrm{Adv}}$$

where *w*_graph_, *w*_Balancing_, *w*_Adv_ are the weights of loss graph reconstruction loss, balancing loss, and adversarial loss, respectively. These weights, which are configured to default values of 10, 100, and 0.1 respectively, serve to balance the various loss components and ensure training stability.

### 4.3 SpaMode for Mosaic Integration

For the mosaic integration task—that is, the joint integration of multiple slices with incomplete modalities—SpaMode offers a two‐step solution: principal component (PC) approximation followed by vertical integration. Specifically, consider a scenario with a section S1 where data for two modalities are available, represented by their PC features $X_{M_{1}}^{S_{1}}\in R^{N_{S_{1}}\times D_{M_{1}}}$, $X_{M_{2}}^{S_{1}}\in R^{N_{S_{1}}\times D_{M_{2}}}$​. Adjacent to it is a section S2 where only modality *M*_1_ is available $X_{M_{1}}^{S_{2}}\in R^{N_{S_{2}}\times D_{M_{1}}}$, while modality *M*_2_ is missing. It is assumed that both slices have been processed to share the same principal component feature space. To accurately reconstruct the feature information of missing modalities, SpaMode employs both inference and translation approaches to complementarily approximate the missing principal component features.

For the inference‐based approach, SpaMode first pre‐trained on the complete‐modality section S1 with $X_{M_{1}}^{S_{1}}$, $X_{M_{2}}^{S_{1}}$. Next, SpaMode takes the available modality data from section S2 as input to infer the latent codes, and then uses the decoder to reconstruct the principal component features of the missing modality 2, denoted $X_{M_{2},\mathrm{recon}}^{S_{2}}\in R^{N_{S_{2}}\times D_{M_{2}}}$.

For the translation‐based approach, SpaMode first captures smooth spatial feature patterns by aggregating features from the spatial neighborhood:

$$\;SP_{i}=\mathrm{SpaAgg}\;\left(X_{i},A_{S}\right)=\frac{1}{\left|A_{S}\left(i\right)\right|}\;\sum_{j\in A_{S}\left(i\right)}X_{j}\;$$

SpaMode captures the spatial patterns of the omics modality, *M*_1_ in our example, that is common to both sections S1 and S2:

$$\;{\mathrm{SP}}_{M_{1}}^{S_{1}}=\mathrm{SpaAgg}\left(X_{M_{1}}^{S_{1}},A_{S_{1}}\right)$$

$$\;{\mathrm{SP}}_{M_{1}}^{S_{2}}=\mathrm{SpaAgg}\left(X_{M_{1}}^{S_{2}},A_{S_{2}}\right)$$

Furthermore, SpaMode performs a nearest‐neighbor (NN) search within ${\mathrm{SP}}_{M_{1}}^{S_{1}}$ for ${\mathrm{SP}}_{M_{1}}^{S_{2}}$​ and compile the resulting indices:

$$\mathrm{Indices}=\mathrm{NN}\left({\mathrm{SP}}_{M_{1}}^{S_{1}},{\mathrm{SP}}_{M_{1}}^{S_{2}}\right)$$

where NN(·, ·) denotes the nearest neighbor search process, which is used to find the index of the closest spot in ${\mathrm{SP}}_{M_{1}}^{S_{1}}$ for each spot in ${\mathrm{SP}}_{M_{1}}^{S_{2}}$ within the Euclidean space. SpaMode translates the missing modality for section S2 using the nearest‐neighbor indices and the PC features of the corresponding modality from slice S1:

$$\;X_{M_{2},\mathrm{Trans}}^{S_{2}}=X_{M_{2}}^{S_{1}}\;\left[\mathrm{Indices}\right]$$

To quantify the translation error, SpaMode also applies the translation procedure to the available modality of slice S2. The translation error is then measured as the cosine similarity between the original and the translated features for this modality:

$$\;X_{M_{1},\mathrm{Trans}}^{S_{2}}=X_{M_{1}}^{S_{1}}\;\left[\mathrm{Indices}\right]$$

$$\mathrm{error}=\frac{X_{M_{1}}^{S_{2}}\cdot X_{M_{1},\mathrm{Trans}}^{S_{2}}}{∥X_{M_{1}}^{S_{2}}∥∥X_{M_{1},\mathrm{Trans}}^{S_{2}}∥}\;\;$$

The final approximation of the PC features for the missing modality in section S2 is expressed as a weighted combination of the reconstructed features and the translated features, where the weight is determined by the translation error:

$$\;X_{M_{2},\mathrm{PCA}}^{S_{2}}=\mathrm{error}\cdot X_{M_{2},\mathrm{Trans}}^{S_{2}}+\left(1-\mathrm{error}\right)\cdot X_{M_{2},\mathrm{recon}}^{S_{2}}\;$$

This weighted design is intended to use the translation accuracy to create a complementary blend of the translated and reconstructed features. Consider the extreme cases: when the translated features perfectly match the source features, i.e., $\lim_{X_{M_{1},\mathrm{Trans}}^{S_{2}}\to X_{M_{1}}^{S_{2}}}\mathrm{error}$= 1, the approximated PC features for the missing modality in S2 will consist entirely of these precise translated features. Conversely, if the translation is poor, the approximation will rely solely on the decoder‐reconstructed features. This dynamic weighting mechanism ensures the robustness of the framework, particularly in edge cases where adjacent tissue slices possess unusually high natural biological variation. In such highly divergent scenarios, direct spatial translation becomes unreliable, leading to a low similarity score (error→0). Consequently, the linear combination adaptively shifts its reliance away from the mismatched translated features, defaulting almost entirely to the inference‐based reconstructed features ($X_{M_{2},\mathrm{recon}}^{S_{2}}$) generated by the pre‐trained decoder. This prevents the propagation of misleading spatial patterns across highly variable slices.

After obtaining the approximated principal component features, the encoding task for the slice with the initially missing modality (S2) effectively becomes a standard vertical integration task, which is a core function of the SpaMode framework.

### 4.4 SpaMode for Count Matrix Translation

Similar to the workflow for approximating the PC features of a missing modality, SpaMode can also translate the raw count matrix, denote as *RM*, for that modality. First, a hybrid spatial pattern is modeled by combining the modality features and spatial neighborhood relationships:

$$\;{\mathrm{SP}}_{mix}^{S_{1}}=SpaAgg\left(Concat\left(X_{M_{1}}^{S_{1}},X_{M_{2}}^{S_{1}}\right),A_{S_{1}}\right)$$

$$\;{\mathrm{SP}}_{mix}^{S_{2}}=SpaAgg\left(Concat\left(X_{M_{1}}^{S_{2}},X_{M_{2},PCA}^{S_{2}}\right),A_{S_{2}}\right)$$

Here, *Concat*(·) denotes the concatenation operation. We then identify the k‐nearest data points in section S1 for each data point in section S2 using the k‐nearest neighbor (k‐NN) principle.

$$k-\mathrm{indices}=\mathrm{kNN}\left({\mathrm{SP}}_{\mathrm{mix}}^{S_{1}},{\mathrm{SP}}_{\mathrm{mix}}^{S_{2}}\right)$$

We translate the raw count matrix for the missing modality in slice S2 by averaging the counts from the k‐nearest neighboring data points identified in slice S1:

$$\;RM_{M_{2}}^{S_{2}}=\frac{1}{k}\;\sum_{j=1}^{k}RM_{M_{2}}^{S_{1}}{\left[k-\mathrm{indices}\right]}_{j}\;$$

Through the aforementioned method, SpaMode can reconstruct gene expression profiles within a spatial context that closely resemble those from real sequencing data. This provides an efficient solution to the challenge of partial modality missingness in the joint analysis of multi‐slice spatial multi‐omics data. Concurrently, this approach, which relies on accurate inference from adjacent slices, also presents the potential to reduce the economic cost associated with sequencing consecutive spatial omics slices.

Overall, SpaMode is a general model that integrates spatial multi‐omics joint analysis, mosaic integration, and missing modality translation. By capturing multi‐omics co‐patterns within a spatial context and being compatible with multi‐slice joint analysis and scenarios with missing modalities, SpaMode provides crucial insights and tools for more comprehensive and in‐depth spatial multi‐omics research.

## Funding

This work was supported by the National Natural Science Foundation of China (Grant No. 32300554, 32370711), the Guangdong Provincial Key Laboratory of Mathematical and Neural Dynamical Systems (Grant No. 2024B1212010004), Shenzhen Medical Research Fund (Grant No.: A2303033); and Shenzhen People's Hospital Physician Scientist Training “Five Three Program” (Grant No.: SYWGSJCYJ202401).

## Conflicts of Interest

The authors declare no conflicts of interest.

## Data and Code Availability

The SpaMode framework is implemented in Python and is open‐source. The complete source code and detailed tutorials for this study are freely available at GitHub (https://github.com/bridge1924/SpaMode). No new experimental datasets were generated during this study. All datasets analyzed during this study are publicly accessible: the Human Lymph Node dataset is available in the Gene Expression Omnibus under accession number GSE263617; the Human Tonsil dataset is hosted on Zenodo (https://zenodo.org/uploads/12654113); the Mouse Brain dataset can be accessed via AtlasXplore (https://web.atlasxomics.com/visualization/Fan/); the mouse embryonic MISAR‐seq dataset is deposited in the National Genomics Data Center under accession number OEP003285; and the ccRCC spatial metabolomics dataset is available on Zenodo (https://zenodo.org/records/14986870). The mouse neocortex single‐cell RNA‐seq reference dataset is available in the Gene Expression Omnibus under accession number GSE161690.

## Supporting information




**Supporting File**: advs75478‐sup0001‐Figure S1‐S8.pdf.

## Data Availability

Data sharing not applicable to this article as no datasets were generated or analyzed during the current study.
