# Supplementary material for: SpaMode: A Broadly Applicable Framework for Deciphering Spatial Multi‐Omics Using Multimodal Mixture of Disentangled Experts
Source: Adv Sci (Weinh). 2026 May 4;13(41):e75478. doi: 10.1002/advs.75478 (PMC13335753; doi:10.1002/advs.75478)
Supplement: Supplementary file 1 — Supporting File: advs75478‐sup0001‐Figure S1‐S8.pdf. [file ADVS-13-e75478-s001.pdf]

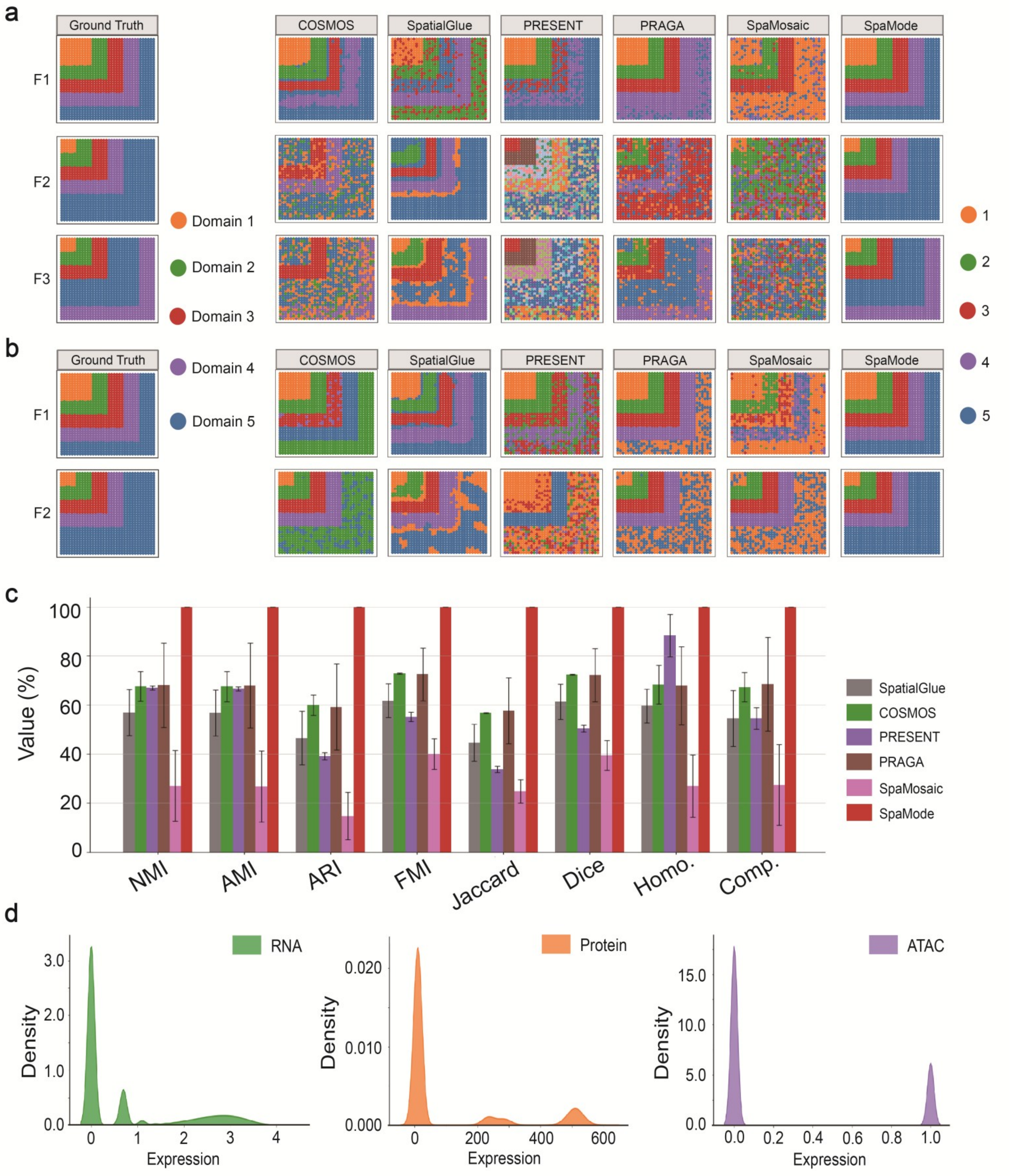

Supplementary Fig. 1 Supplementary results for the simulated dataset. (a-b) Visualization of clustering results from SpaMode and benchmark methods on the simulated RNA-ADT **(a)** and RNA-ATAC **(b)** datasets. **(c)** Quantitative comparisons on the simulated RNA-ADT dataset across three sections. **(d)** Statistical distributions of the simulated RNA, protein, and ATAC sequencing data.

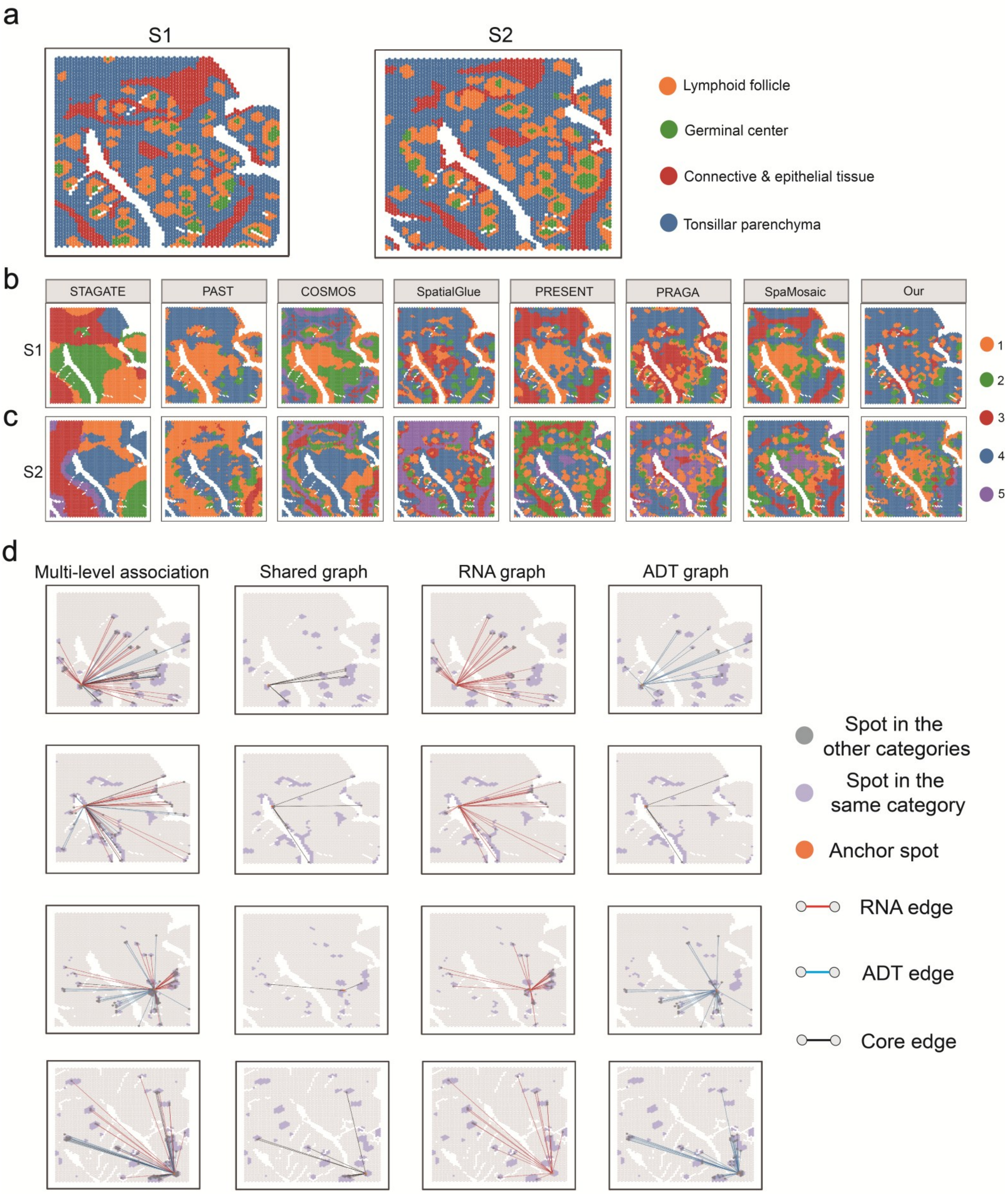

Supplementary Fig. 2 Supplementary results for the human tonsil dataset. (a) Ground truth for the human tonsil dataset S1 and S2 sections. **(b-c)** Visualization of spatial domain identified by SpaMode and baseline methods on the S1 **(b)** and S2 **(c)** sections. **(d)** Spatial visualization of the multi-level association network, shared graph, RNA-specific graph, and ADT-specific graph on the human tonsil dataset.

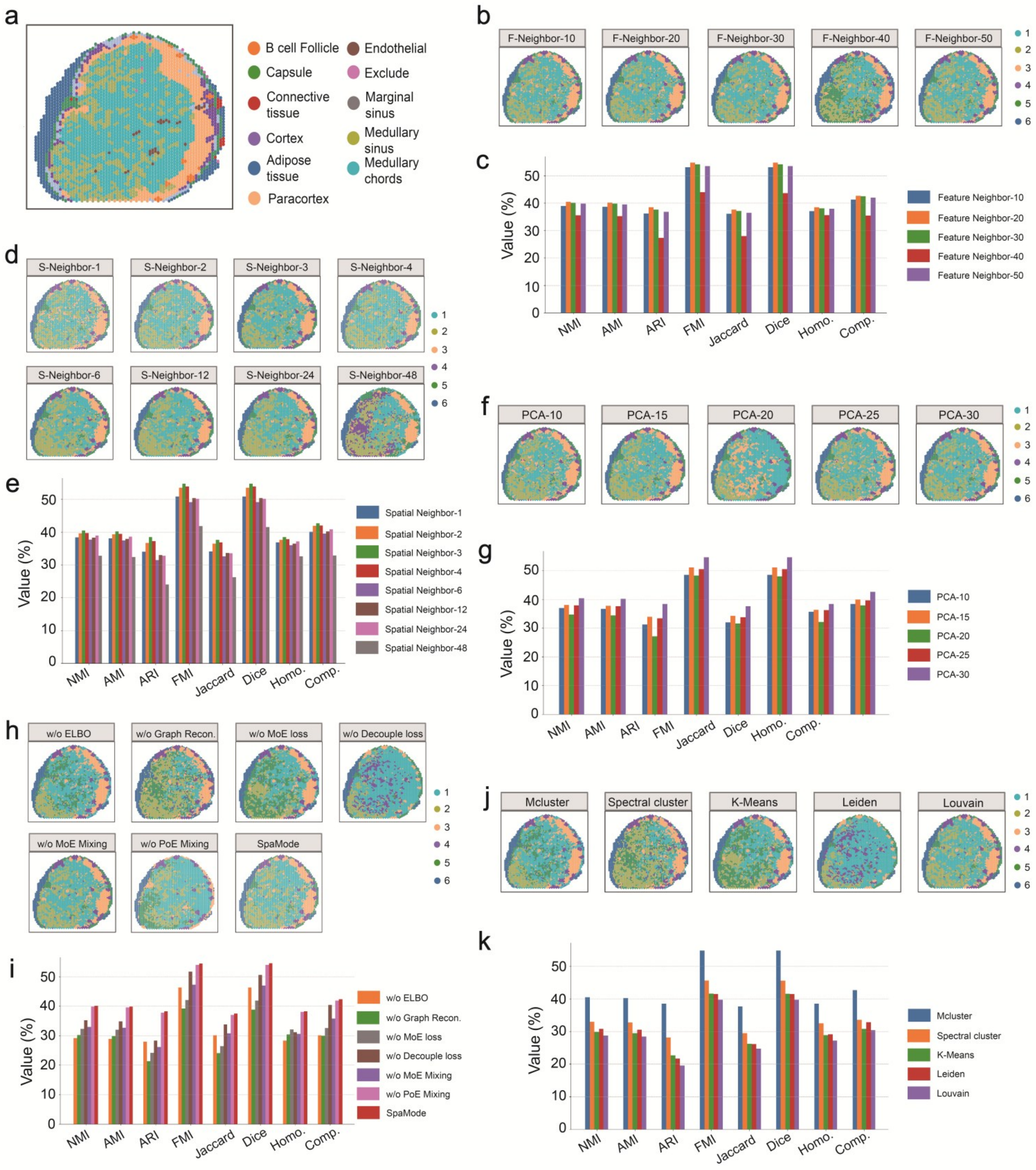

Supplementary Fig. 3 Parameter sensitivity and ablation studies on the human lymph node D1 section. **(a)** Ground truth annotations for the D1 section. **(b-g)** Parameter sensitivity analysis showing clustering visualizations and quantitative metrics for varying numbers of feature neighbors **(b-c)**, spatial neighbors **(d-e)**, and principal components **(f-g)**. **(h-i)** Ablation study comparing SpaMode variations in terms of spatial domains **(h)** and quantitative metrics **(i)**. **(j-k)** Performance comparison of different clustering algorithms within the SpaMode framework.

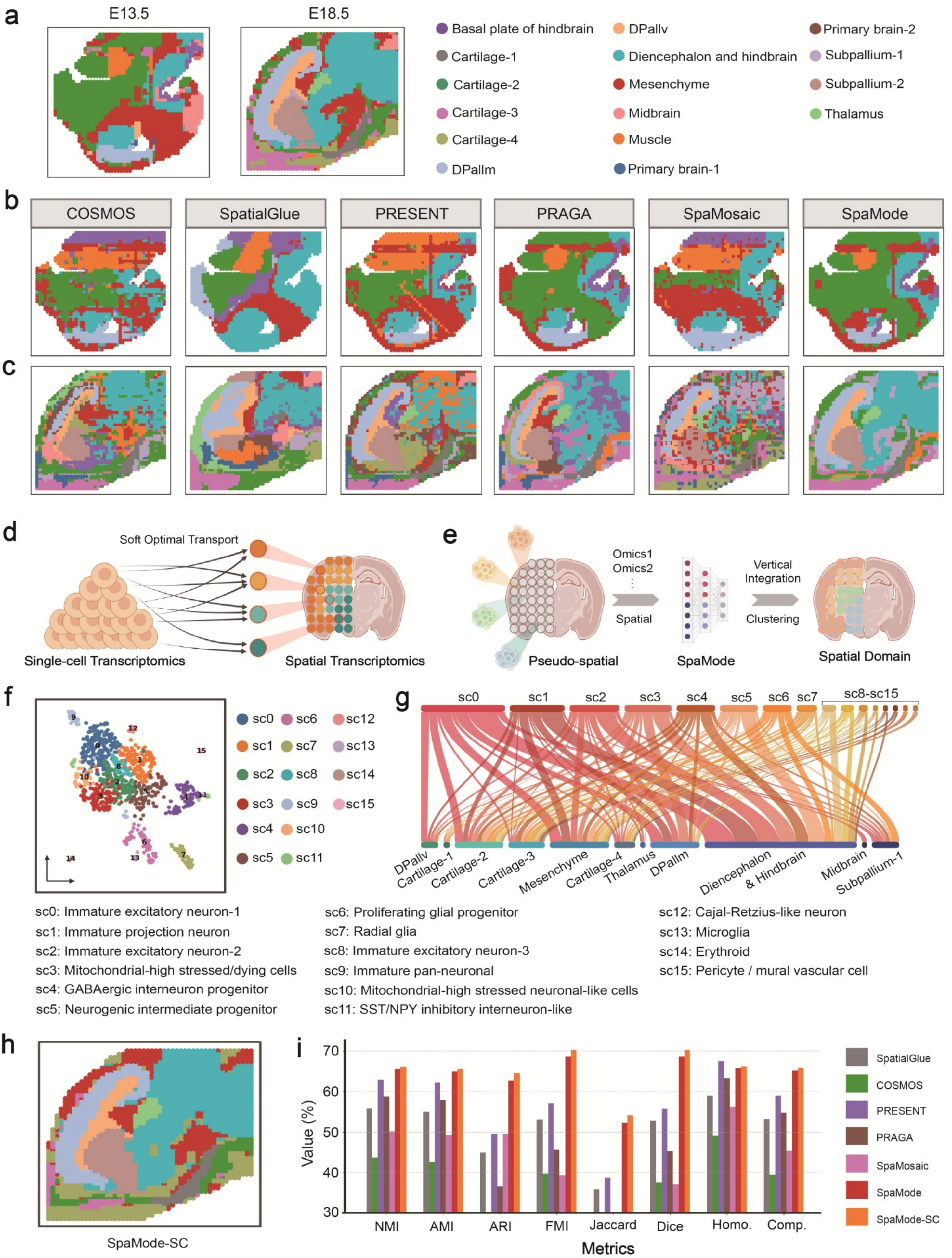
a
E13.5
E18.5
Basal plate of hindbrain
Cartilage-1
Cartilage-2
Cartilage-3
Cartilage-4
DPallm
DPallv
Diencephalon and hindbrain
Mesenchyme
Midbrain
Muscle
Primary brain-1
Primary brain-2
Subpallium-1
Subpallium-2
Thalamus
b
COSMOS
SpatialGlue
PRESENT
PRAGA
SpaMosaic
SpaMode
c
d
Soft Optimal Transport
Single-cell Transcriptomics
Spatial Transcriptomics
e
Omics1
Omics2
Spatial
Vertical Integration
Clustering
Pseudo-spatial
SpaMode
Spatial Domain
f
sc0 sc1 sc2 sc3 sc4 sc5 sc6 sc7 sc8 sc9 sc10 sc11 sc12 sc13 sc14 sc15
g
sc0 sc1 sc2 sc3 sc4 sc5 sc6 sc7 sc8-sc15
DPallv
Cartilage-1
Cartilage-2
Cartilage-3
Mesenchyme
Cartilage-4
Thalamus
DPallm
Diencephalon & Hindbrain
Midbrain
Subpallium-1
sc0: Immature excitatory neuron-1
sc1: Immature projection neuron
sc2: Immature excitatory neuron-2
sc3: Mitochondrial-high stressed/dying cells
sc4: GABAergic interneuron progenitor
sc5: Neurogenic intermediate progenitor
sc6: Proliferating glial progenitor
sc7: Radial glia
sc8: Immature excitatory neuron-3
sc9: Immature pan-neuronal
sc10: Mitochondrial-high stressed neuronal-like cells
sc11: SST/NPY inhibitory interneuron-like
sc12: Cajal-Retzius-like neuron
sc13: Microglia
sc14: Erythroid
sc15: Pericyte / mural vascular cell
h
SpaMode-SC
i
Value (%)
70
60
50
40
30
NMI AMI ARI FMI Jaccard Dice Homo. Comp.
Metrics
SpatialGlue
COSMOS
PRESENT
PRAGA
SpaMosaic
SpaMode
SpaMode-SC

Supplementary Fig. 4 Supplementary results for the mouse embryonic dataset. (a) Ground truth for the mouse embryonic dataset E13.5 and E18.5. **(b)** Visualization of spatial domain clustered by SpaMode and baseline methods on the mouse embryonic E13.5 data. **(c)** Visualization of spatial domain clustered by SpaMode and baseline methods on the mouse embryonic E18.5 data. **(d)** Schematic illustration of mapping single-cell transcriptomic profiles to spatial transcriptomics spots using soft optimal transport. **(e)** Workflow of constructing a pseudo-spatial modality from the mapped single-cell data and vertically integrating it with existing spatial multi-omics layers using SpaMode. **(f)** UMAP visualization of the E18.5 mouse neocortex single-cell reference dataset, annotated into distinct cell populations (sc0-sc15). **(g)** Correspondence plot illustrating the alignment and mapping weights between single-cell clusters and deciphered spatial domains, guided by the optimal transport matrix. **(h)** Visualization of spatial domains deciphered by SpaMode-SC (SpaMode enhanced with single-cell reference integration) on the E18.5 section. **(i)** Quantitative comparison of clustering performance across eight evaluation metrics among baseline methods, standard SpaMode, and SpaMode-SC on the E18.5 dataset.

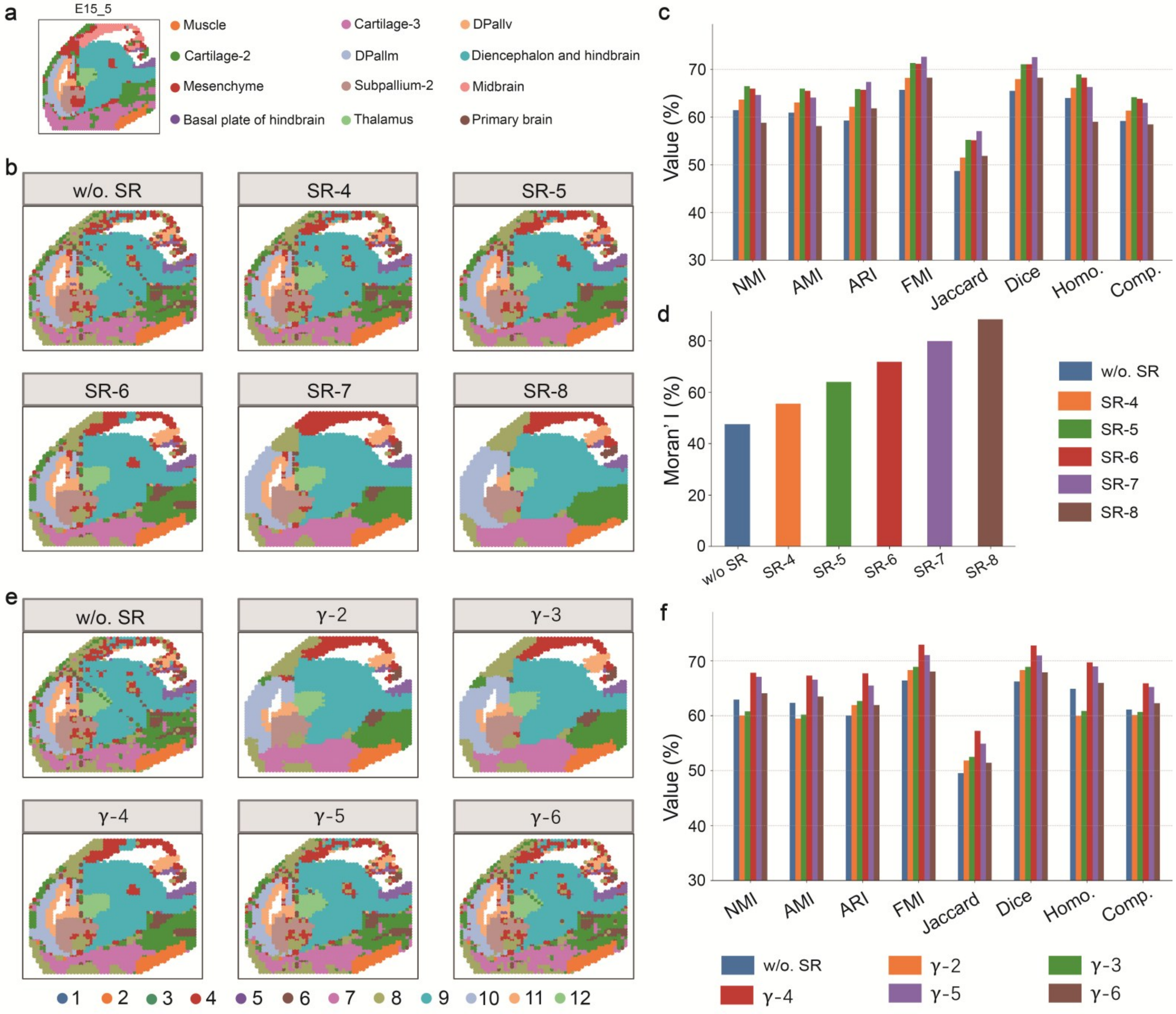

Supplementary Fig. 5 Validation results of spatial rectification for the mouse embryonic dataset E15.5. (a) Ground truth for the mouse embryonic dataset E13.5. **(b)** Visualization results for different spatial rectification ranges (SR). **(c)** Quantitative results for different spatial rectification ranges. **(d)** Moran's index for different spatial rectification ranges. **(e)** Visualization results for different correction thresholds (γ). **(f)** Quantitative results for different correction thresholds (γ)

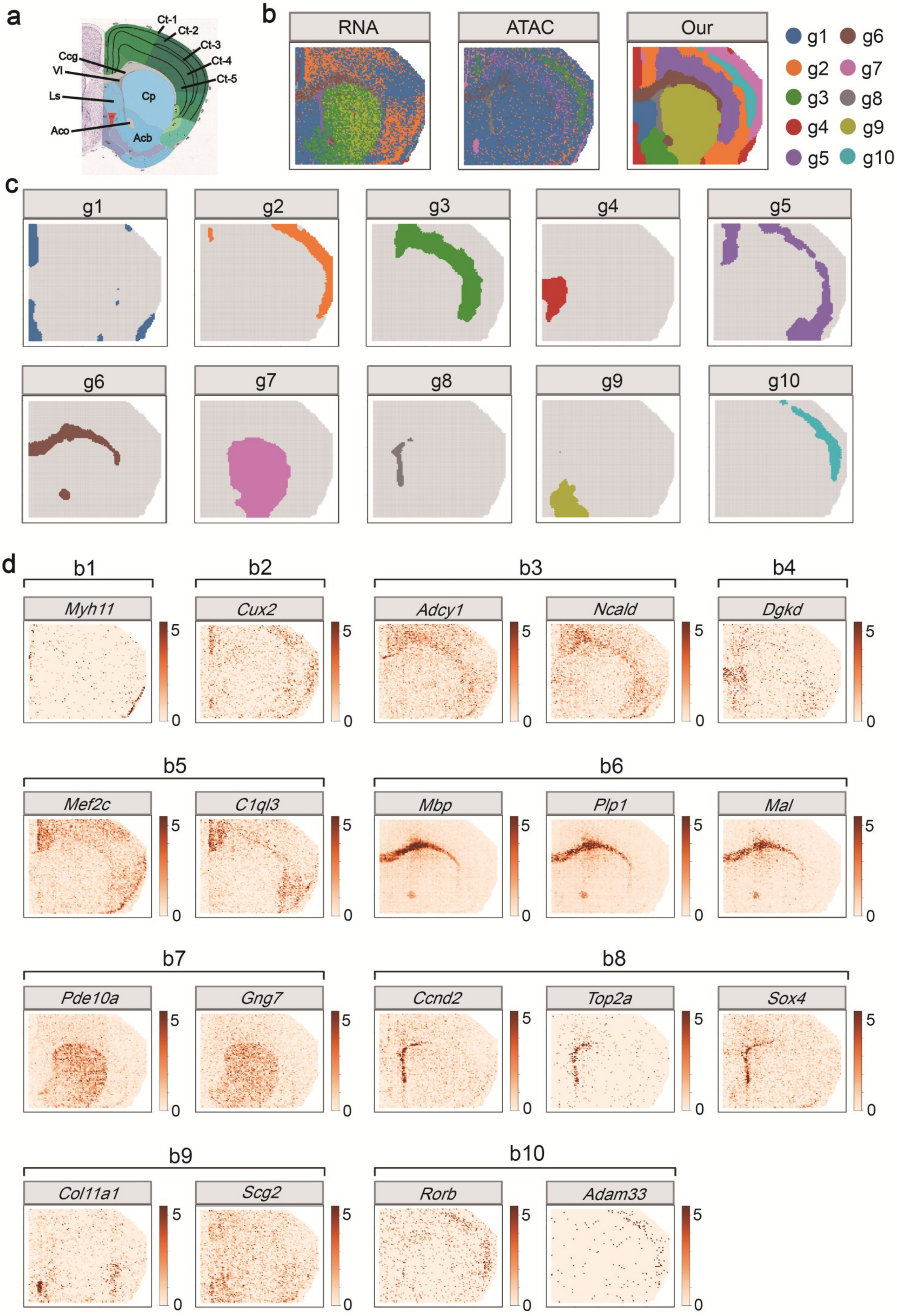

a
Ct-1
Ct-2
Ct-3
Ct-4
Ct-5
Ccg
Vl
Ls
Cp
Aco
Acb
b
RNA
ATAC
Our
g1 g2 g3 g4 g5 g6 g7 g8 g9 g10
c
g1 g2 g3 g4 g5
g6 g7 g8 g9 g10
d
b1 Myh11
b2 Cux2
b3 Adcy1 Ncald
b4 Dgkd
b5 Mef2c C1ql3
b6 Mbp Plp1 Mal
b7 Pde10a Gng7
b8 Ccnd2 Top2a Sox4
b9 Col11a1 Scg2
b10 Rorb Adam33
5
0

Supplementary Fig. 6 Supplementary results for the mouse brain dataset. (a) Annotation of mouse brain regions with reference to the Allen Brain Atlas. **(b)** Visualization of spatial domain clustered by RNA, ATAC, and SpaMode. **(c)** Visualization of each domain in SpaMode clustering. **(d)** Spatial expression patterns of marker genes in each spatial domain selected by SpaMode.

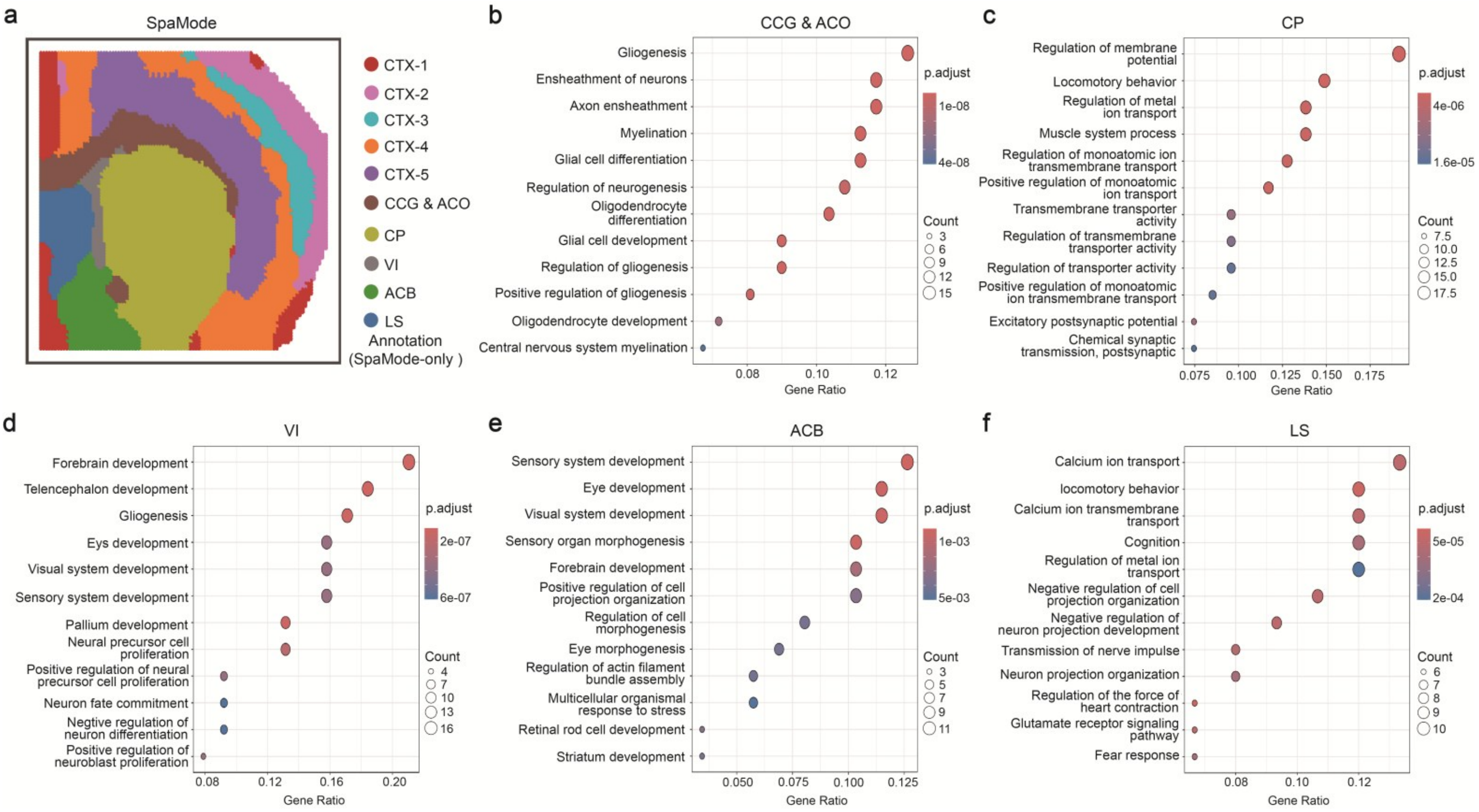

Supplementary Fig. 7 Biological interpretation of spatial domains identified by SpaMode in the mouse brain dataset. **(a)** Spatial visualization of fine-grained brain regions identified and annotated solely by SpaMode (SpaMode-only annotation), distinct from the coarse-grained annotations. Regions include layers of the Cortex (CTX-1 to CTX-5), Genu of the corpus callosum & Anterior commissure (CCG & ACO), Caudoputamen (CP), Ventral areas (VI), Nucleus accumbens (ACB), and Lateral septal complex (LS). **(b-f)** Dot plots of Gene Ontology (GO) enrichment analysis for representative spatial domains, validating their biological identities. The plots show the top enriched biological processes for: **(b)** CCG & ACO, highlighting myelination and glial cell development; **(c)** CP, highlighting locomotory behavior and regulation of membrane potential; **(d)** VI, highlighting forebrain development and neural precursor cell proliferation; **(e)** ACB, highlighting sensory system development and morphogenesis; **(f)** LS, highlighting fear response and calcium ion transport. The size of the dots represents the gene count, and the color gradient represents the adjusted p-value.

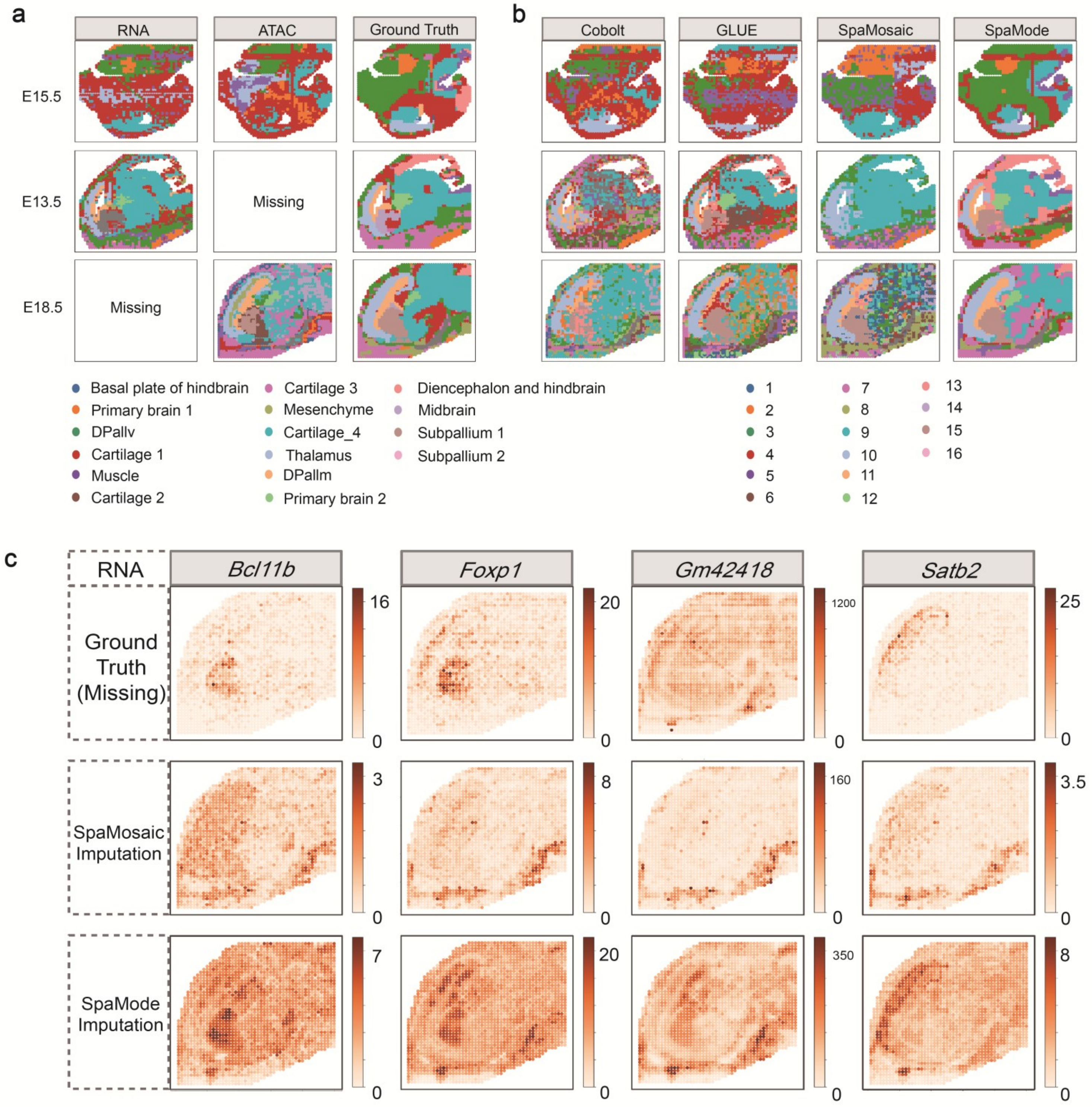

Supplementary Fig. 8 Supplementary Mosaic integration results for the mouse embryonic dataset. (a) Ground truth for the mouse embryonic dataset, assuming all omics modalities are available for E13.5, while only the RNA modality is available for E15.5, and only the ADT modality is available for slice E18.5. **(b)** Visualization of spatial domain clustering by baseline methods and SpaMode on the mouse embryonic dataset under a setting of partial modality missing. **(c)** Comparison of spatial expression patterns and quantitative metrics for the ground truth protein count matrix versus the protein count matrices inferred by SpaMosaic and SpaMode on the mouse embryonic dataset.
